# Macrophage-based delivery of interleukin-13 improves functional and histopathological outcomes following spinal cord injury

**DOI:** 10.1186/s12974-022-02458-2

**Published:** 2022-04-29

**Authors:** Jana Van Broeckhoven, Céline Erens, Daniela Sommer, Elle Scheijen, Selien Sanchez, Pia M. Vidal, Dearbhaile Dooley, Elise Van Breedam, Alessandra Quarta, Peter Ponsaerts, Sven Hendrix, Stefanie Lemmens

**Affiliations:** 1 Department of Immunology and Infection, Biomedical Research Institute, Hasselt University, 3590 Diepenbeek, Belgium; 2 Department of Neurosciences, Biomedical Research Institute, Hasselt University, 3590 Diepenbeek, Belgium; 3 Neuroimmunology and Regeneration of the Central Nervous System Unit, Biomedical Science Research Laboratory, Basic Sciences Department, Faculty of Medicine, Universidad Católica de la Santísima Concepción, 4090541 Concepción, Chile; 4 School of Medicine, Health Sciences Centre, University College Dublin, Belfield, Dublin 4, Ireland; 5 UCD Conway Institute of Biomolecular & Biomedical Research University College Dublin, Belfield, Dublin 4, Ireland; 6 Laboratory of Experimental Hematology, University of Antwerp, 2610 Wilrijk, Belgium; 7 Vaccine and Infectious Disease Institute (Vaxinfectio), University of Antwerp, 2610 Wilrijk, Belgium; 8 Medical School Hamburg, Am Kaiserkai 1, 20457 Hamburg, Germany

**Keywords:** CNS trauma, Neuroinflammation, Macrophages, Immunomodulation, Interleukin-13, Neurospheroids

## Abstract

**Background:**

Spinal cord injury (SCI) elicits a robust neuroinflammatory reaction which, in turn, exacerbates the initial mechanical damage. Pivotal players orchestrating this response are macrophages (Mφs) and microglia. After SCI, the inflammatory environment is dominated by pro-inflammatory Mφs/microglia, which contribute to secondary cell death and prevent regeneration. Therefore, reprogramming Mφ/microglia towards a more anti-inflammatory and potentially neuroprotective phenotype has gained substantial therapeutic interest in recent years. Interleukin-13 (IL-13) is a potent inducer of such an anti-inflammatory phenotype. In this study, we used genetically modified Mφs as carriers to continuously secrete IL-13 (IL-13 Mφs) at the lesion site.

**Methods:**

Mφs were genetically modified to secrete IL-13 (IL-13 Mφs) and were phenotypically characterized using qPCR, western blot, and ELISA. To analyze the therapeutic potential, the IL-13 Mφs were intraspinally injected at the perilesional area after hemisection SCI in female mice. Functional recovery and histopathological improvements were evaluated using the Basso Mouse Scale score and immunohistochemistry. Neuroprotective effects of IL-13 were investigated using different cell viability assays in murine and human neuroblastoma cell lines, human neurospheroids, as well as murine organotypic brain slice cultures.

**Results:**

In contrast to Mφs prestimulated with recombinant IL-13, perilesional transplantation of IL-13 Mφs promoted functional recovery following SCI in mice. This improvement was accompanied by reduced lesion size and demyelinated area. The local anti-inflammatory shift induced by IL-13 Mφs resulted in reduced neuronal death and fewer contacts between dystrophic axons and Mφs/microglia, suggesting suppression of axonal dieback. Using IL-4Rα-deficient mice, we show that IL-13 signaling is required for these beneficial effects. Whereas direct neuroprotective effects of IL-13 on murine and human neuroblastoma cell lines or human neurospheroid cultures were absent, IL-13 rescued murine organotypic brain slices from cell death, probably by indirectly modulating the Mφ/microglia responses.

**Conclusions:**

Collectively, our data suggest that the IL-13-induced anti-inflammatory Mφ/microglia phenotype can preserve neuronal tissue and ameliorate axonal dieback, thereby promoting recovery after SCI.

**Supplementary Information:**

The online version contains supplementary material available at 10.1186/s12974-022-02458-2.

## Background

Following spinal cord injury (SCI), a wide variety of secondary injury processes are provoked, which significantly worsens patients’ outcomes [1]. Among these, the most complex and important one is the neuroinflammatory response that contributes to progressive neurodegeneration. Macrophages (Mφs) and microglia are one of the key players, with a predominant role for Mφs, which accumulate specifically at the lesion epicenter [1, 2]. At the injury site, local factors (e.g., cytokines, myelin debris, and apoptotic cells) determine the phenotypic properties of these cells, rendering them either detrimental or beneficial for disease pathology [1, 2]. A general pragmatic classification divides them into (i) classically activated M1 Mφs, which secrete pro-inflammatory factors and induce axonal dieback, and (ii) alternatively activated M2 Mφs, which display anti-inflammatory properties and support axonal regrowth [3, 4]. The M1/M2 dichotomy is an oversimplified model as Mφs are very heterogenic with remarkable functional plasticity [5]. Nevertheless, the M1/M2 paradigm helped to identify significant functional characteristics of selected Mφ populations.

In particular, M2a Mɸs have been identified as essential players in neuroregeneration by counteracting inflammation and promoting wound healing [6, 7]. This subset is characterized by upregulation of the signature molecules Arginase-1 (Arg1), found in inflammatory zone 1 (FIZZ1), chitinase-3-like protein 3 (Chi3l3, Ym1), and mannose receptor (CD206) [8, 9]. This M2 Mφ subpopulation arrives first at the lesion site in the acute phase after injury but is only transiently detectable [3]. After SCI, the perilesional milieu favors M1 polarization, resulting in a persistently hostile environment for repair [1, 3]. As this is a major contributor to poor regeneration, reducing the M1 dominance and promoting an M2 presence has become an attractive therapeutic approach for SCI.

In this study, the goal was to modulate perilesional Mφs/microglia and specifically exploit their M2a phenotype to influence motor recovery beneficially. For this aim, we used the canonical T-helper cell type 2 cytokine interleukin-13 (IL-13), which is one of the main inducers of this M2a subset [9]. Following SCI, IL-13 level dramatically decreases in the spinal cord tissue [10, 11]. This drop suggests that the application of IL-13 in the acute phase after SCI may have therapeutic potential by altering Mφ/microglia polarization. Previous animal studies in the context of central nervous system (CNS) trauma revealed that IL-13 administration, either directly or via cell therapy, exerted anti-inflammatory effects and thereby provided neuroprotection [12–14]. Our prior study demonstrated that cell-based delivery of IL-13 via mesenchymal stem cells (MSCs) enhanced functional recovery after SCI. This improvement was associated with an increased perilesional presence of Arg1^+^ Mφs/microglia. Thus, it was tempting to speculate that accumulated M2a Mφs were critical for functional repair [15]. Therefore, in this study, we used Mφs to continuously deliver IL-13 as (i) Mφs have a high migratory capacity towards inflammatory sites after trauma and (ii) IL-13 can introduce additional anti-inflammatory M2a Mφs at the lesion site [16].

We show for the first time that the use of genetically modified Mφs as carriers of IL-13 is a potent strategy to improve histopathological and functional recovery after SCI. Using IL-4Rα-deficient mice, we demonstrate that IL-13 signaling is essential to reduce neuronal death and suppress axonal dieback by promoting an anti-inflammatory phenotypic shift of perilesional Mφs/microglia. Overall, these findings underline the therapeutic potential of IL-13 and Mφ/microglia modulation in regeneration after CNS trauma.

## Methods

### Animals

In vitro experiments were done using female 8–12-week old wild type (WT) BALB/c (Envigo) or C57BL/6J mice (Janvier Labs). CX3CR1^+^/GFP mice were kindly provided by prof. Brône (Uhasselt). In vivo experiments were performed with either female 10–14-week old WT C57BL/6j mice (Janvier Labs) or IL-4Rα control and knockout (KO) BALB/cJ mice (#000651, #003514, The Jackson Laboratory) [17]. As male mice have a substantially higher dropout rate after SCI due to wounding and higher risk of bladder or other infections, only female mice were used in this study. Mice were housed at the conventional animal facility of Hasselt University under stable conditions (temperature-controlled room, 12 h light/dark cycle, food, and water ad libitum). Experiments and sample size calculations were approved by the local ethical committee and were performed according to the guidelines of Directive 2010/63/EU on the protection of animals used for scientific purposes.

### Macrophage culture

Bone-marrow-derived Mɸs were obtained from female mice. In brief, primary bone marrow cells were isolated by flushing the femur and tibia with 1× phosphate-buffered saline (PBS, Lonza). Cells were cultured in RPMI 1640 medium (Lonza) supplemented with 10% heat-inactivated fetal calf serum (FCS, Gibco), 1% penicillin/streptomycin (100 units/ml penicillin, 100 μg/ml streptomycin, Invitrogen), and 15% L929 conditioned medium at 37 °C and 5% CO_2_. Every 2–3 days, partial or complete medium change was done. After 7 days, naive M0 Mɸs were obtained. To induce M1 or M2 polarization, Mɸs were stimulated with 200 ng/ml lipopolysaccharide (LPS, Merck) for 24 h or 33.3 ng/ml recombinant IL-13 (rIL-13, PeproTech) for 48 h, respectively. Conditioned medium (CM) was collected after stimulation.

### Lentiviral transduction

To generate IL-13 overexpressing and Arg1 overexpressing Mɸs, naive Mɸs were transduced with pIRES-mIL-13-puro or pIRES-Arg1-puro lentiviral particles provided by the Laboratory for Viral Vector Technology & Gene Therapy (Leuven Viral Vector Core of the KU Leuven) according to previously optimized protocol with minor adaptations [15]. Mɸs were seeded in a 24-well plate at 1.5 × 10^5^ cells/well in standard medium. Positive selection of the transduced cells was made using puromycin (2.25 μg/ml, InvivoGen) for 72 h. CM was collected after puromycin selection.

### qPCR

Cells were lysed using QIAzol (Qiagen), and RNA was extracted using the RNeasy mini kit (Qiagen), according to the manufacturer’s instructions. Conversion to cDNA was done with qScript cDNA supermix (Quanta Biosciences). Quantitative PCR was performed on a StepOnePlus detection system (Applied Biosciences) under universal cycle conditions. Primer sequences can be found in the Additional information (Additional file 1: Table S1). The comparative Ct method was used to determine relative gene expression. Data were normalized to the most stable housekeeping genes (CYCA, GADPH, HMBS, HPRT, YHWAZ) determined by GeNorm.

### Western blot

Cells were lysed using RIPA buffer supplemented with protease and phosphatase inhibitors (Sigma-Aldrich). The protein concentration was determined by the Pierce™ BCA assay kit (ThermoFisher Scientific), according to the manufacturer's guidelines. Samples (4–10 µg) were separated on 7.5% or 12% (dependent on size target) sodium dodecyl sulfate–polyacrylamide gels by electrophoresis (1 h at 200 V) and transferred to a polyvinylidene fluoride membrane (1.5 h at 350 mA, Merck). After blocking using 5% non-fat dry milk in 1× tris-buffered saline supplemented with 0.05% Tween 20 (TBS-T), blots were incubated with primary antibodies overnight at 4 °C. Used antibodies included: mouse anti-Arg1 (1:1000, sc-271430, Santa Cruz Biotechnology), mouse anti-inducible nitric oxide synthase (iNOS, 1:500, N6657, Sigma-Aldrich), and mouse anti-β-actin (1:2000, sc-47778, Santa Cruz). After washing, secondary antibodies (anti-mouse HRP, 1:2000, Dako) were applied for 1 h at RT (room temperature). β-Actin was used as a loading control. Visualization was done using the Pierce™ ECL plus Western blotting substrate (ThermoFisher Scientific). Images were taken with an Amersham Imager 680 (GE Healthcare Bio-Sciences) and analyzed using the ImageQuant TL software. Pictures displayed in the figures were digitally enhanced to improve visibility.

### ELISA

IL-4 and IL-13 secretion in the supernatants of cell cultures was measured using ELISA kits (ThermoFisher Scientific), according to the manufacturer’s instructions.

### Griess assay

Nitrite secretion in the supernatants of cell cultures was measured using the Griess reagent system kit (Promega), according to the manufacturer’s instruction.

### Spinal cord injury model

A T-cut spinal cord hemisection injury was performed as previously described [15, 18]. Briefly, mice were anesthetized with 2–3% isoflurane (IsofFlo, Abbot Animal Health) and underwent a partial laminectomy at thoracic level 8. Iridectomy scissors were used to transect left and right dorsal funiculi, the dorsal horns, and the ventral funiculus, resulting in a T-cut hemisection injury. The back muscles were sutured, and the skin was closed with wound clips (Autoclip, Clay-Adams Co. Inc.). Post-operative treatment included blood-loss compensation by glucose and pain relief by buprenorphine (0.1 mg/kg bodyweight, Temgesic). Mice were placed in a recovery chamber (33 °C) until they regained consciousness. Bladders were voided daily until animals were able to urinate independently.

### Cell transplantation

For in vivo transplantation, Mφs were isolated from the same mouse strain as the recipient mice in the in vivo experiment, being either C57BL/6J or BALB/c. Mφs were cultured using Nunc™ Dishes with UpCell™ Surface (ThermoFisher Scientific) to avoid enzymatic harvesting. Immediately after SCI induction, mice were randomized and received an intraspinal injection of vehicle or (GFP^+^) Mφs. As previously described, a motorized stereotaxic injector pump (Stoelting) with a 10 µl Hamilton syringe (34-gauge needle) was positioned 1–3 mm rostral to the lesion [19]. The needle was stereotactically inserted into the spinal cord at a depth of 1 mm. Either vehicle (PBS or RMPI) or cells (1 × 10^4^ in 2.5 µl) were injected over a 4 min period, after which the needle was kept in place for again 4 min to allow pressure equilibration and prevent backflow. Investigators were blinded to the treatment groups during the complete experiment.

### Locomotion testing

Functional recovery was assessed using the standardized Basso Mouse Scale (BMS) for locomotion (0 = complete hind limb paralysis, 9 = normal motor function) [20]. Scores are based on hind limb movements in an open field during a 4 min interval. At the day of operation, animals were checked for locomotion, and no abnormalities were observed (BMS score = 9). The evaluation was done by an investigator blinded to the experimental groups and was performed daily from 1 until 7 days post-injury (dpi), followed by a scoring every 2nd or 3rd day. For the analysis, the mean BMS score of the left and right hind limb was used for each animal. Mice who did not display any increase in BMS score were excluded.

### Immunohistochemical analysis

At 8 or 28 dpi, mice received an overdose of dolethal (Vetiquinol B.V.) and were transcardially perfused using ringer solution supplemented with heparin, followed by perfusion with 4% paraformaldehyde (PFA) in PBS. Longitudinal cryosections of 10 µm were obtained. Immunofluorescent stainings were done as previously described [15, 18].

To determine the presence of GFP^+^ cells, sections were only counterstained with DAPI (1:1000, Sigma-Aldrich) for 10 min. To investigate lesion size, demyelinated area, astrogliosis, and immune cell infiltration, sections were blocked using 10% protein block (Dako) in PBS containing 0.5% Triton-X-100 for 1 h at RT. Primary antibodies included mouse anti-glial fibrillary acidic protein (GFAP, 1:500, G3893, Sigma-Aldrich), rat anti-myelin basic protein (MBP, 1:250, MAB386, Merck), rabbit anti-Iba-1 (1:350, 019-19741, Wako), goat anti-ionized calcium binding adaptor molecule 1 (Iba-1, 1:250, NB100-1028, Novus Biologicals), rabbit anti-neurofilament (NF, 1:100, MA5-14981, ThermoFisher Scientific), mouse anti-NeuN (1:1000, MAB377, Merck), rabbit anti-cleaved caspase 3 (1:100, 9661, Cell Signaling) were diluted in PBS with 1% protein block and 0.5% Triton-X-100 and were incubated overnight at 4 °C. To investigate Arg1^+^ and major histocompatibility complex class ll (MHCII)^+^ cells, an extra permeabilization step using 0.1% Triton-X-100 in TBS for 30 min was performed before blocking the sections for 1 h with 10% protein block in TBS. Primary antibodies, goat anti-Arg1 (1:50, sc-18354, Santa Cruz) and rat anti-MHCII (1:200, sc-59322, Santa Cruz), were dissolved in TBS containing 10% non-fat dry milk powder and were incubated overnight at 4 °C. Following washing, secondary antibody incubation was done for 1 h at RT. The following secondary antibodies were used: goat anti-rat Alexa fluor 488 (1:250, A11006, ThermoFisher Scientific), goat anti-mouse Alexa fluor 568 (1:250, A11004, ThermoFisher Scientific), goat anti-mouse GaM IgG1 fluor 488 (1:400, A21121, Invitrogen), donkey anti-rabbit IgG 555 (1:250, A31572, Invitrogen), goat anti-rabbit Alexa fluor 488 (1:250, A11008, ThermoFisher Scientific), donkey anti-goat Alexa fluor 488 (1:400, A11055, ThermoFisher Scientific) and donkey anti-goat Alexa fluor 555 (A21432, ThermoFisher Scientific). The specificity of secondary antibodies was tested by omitting the primary antibody. Counterstaining with DAPI was performed for 10 min. Pictures were taken using a Nikon Eclipse 80i fluorescence microscope and analyzed with NIS-Elements Viewer 4.0 software or using a LEICA DM4000 B LED microscope and LAS X software (Leica Microsystems).

To investigate lipid load, an oil red O (ORO) staining was performed. Sections were incubated with a 0.33% ORO solution (Sigma-Aldrich) for 15 min, followed by a 5 min incubation with 60% isopropanol. To wash away any unbound particles, the sections were rinsed with distilled water. Pictures were taken using the Leica DM 2000 LED microscope (Leica Microsystems).

### Immunohistochemical quantification

Quantification was carried out on the original, unedited pictures. Pictures displayed in the figures were digitally enhanced to improve visibility. Quantification of histopathological parameters was performed as previously described and was done by investigators blinded to experimental groups [15, 18]. To investigate cell survival, the number of GFP^+^ cells at the injection, perilesional, and lesion site were counted. To quantify lesion size (GFAP^−^ area) and demyelinated area (MBP^−^ area), 5–7 sections per animal containing lesion center and consecutive rostral and caudal area were analyzed. The proximal and distal end of the lesion was detected by identifying the area, where the integrity of the dorsal side of the spinal cord tissue is disrupted. As no intensity was measured, contrast was altered differently among groups. To determine astrogliosis (GFAP expression) and Mφ/microglia presence (Iba-1 expression or Iba-1^+^ cells), an intensity analysis was performed, or the number of positive cells was counted using ImageJ [15]. To identify alternatively and classically activated phagocytes, Arg1^+^ or MHCII^+^ cells were counted caudally and rostrally from the lesion site. Quantification of foam cell presence was carried out using ImageJ software according to the method previously described by Deutsch et al. with slight adaptations [21]. To determine neuronal cell death at the lesion site, the number of cleaved caspase 3^+^NeuN^+^ cells was counted at both rostral and caudal areas from the lesion epicenter and normalized to the amount of NeuN^+^ cells. To quantify the interaction between axons and Mφ/microglia, the number of contacts between NF^+^ dystrophic axons and Iba-1^+^ cells was counted in the perilesional environment. Of note, dystrophic axons were identified by their globular and bulbus morphology extending from an axon fiber [22, 23]. A contact was detected when a cell–cell interaction between a dystrophic axonal bulb and an Iba-1^+^ cell contained a DAPI nucleus. For analysis, two standardized areas, rostral and caudal, from the lesion epicenter were used. The mean number of contacts in these two areas per animal was quantified.

### Neuroblastoma cell culture

The murine Neuro2A cell line (CCL-131™, ATCC®) was cultured in RPMI 1640 medium supplemented with 10% FCS and 1% penicillin/streptomycin. The human SH-SY5Y cell line (Sigma-Aldrich) was cultured in DMEM (Gibco) supplemented with 10% FCS and 1% penicillin/streptomycin. Cells were maintained at 37 °C and 5% CO_2_. For immunocytochemistry, cells were seeded in a 24-well plate at 3 × 10^4^ cells/well for 48 h. To perform cell viability assay, cells were seeded in a 96-well plate at 5 × 10^3^ cells/well and left untreated for 24 h to attach.

### Immunocytochemistry

Cells were fixed with 4% PFA and blocked for 1 h at RT with 10% protein block in PBS. Anti-IL-13Rα1 and anti-IL-4Rα (1:250, ab79277, Abcam, and 1:50, NBP1-00884, Novus Biologicals) antibodies were diluted in PBS with 1% protein block and were incubated overnight at 4 °C. Following washing, secondary antibody (donkey anti-rabbit IgG 555, 1:500) incubation was done for 1 h at RT. The specificity of the secondary antibody was tested by omitting the primary antibody. Counterstaining with DAPI was performed for 10 min. Pictures were taken using a LEICA DM4000 B LED microscope and LAS X software (Leica Microsystems).

### Brain slice isolation and culture

Hippocampal entorhinal cortex slices were prepared from 8 days old pups. In short, brains were isolated in dissection medium (MEM, 2 mM l-glutamine, and distilled water, Gibco) and using a tissue chopper (McILWAIN, H. Saur), 350 μm thick slices were made. These were cultured on inserts (0.4 μm porous, Merck) in incubation medium containing 50% MEM, 25% HBSS, 25% heat-inactivated horse serum, 4 mM l-glutamine, 4 ng/ml insulin, 800 ng/ml ascorbic acid, 2.4 mg/ml glucose and 1% penicillin/streptomycin, Gibco) [24]. In addition, varying concentrations of rIL-13 (0, 5, 50, and 500 ng/ml) were added to the medium for 72 h.

### Neurospheroid culture and staining

Neurospheroids were generated by seeding human-induced pluripotent stem cell-derived neural stem cells (hiPSC-NSCs, 1.6 × 10^4^ cells/well), stably expressing luciferase in an ultra-low attachment 96-well plate. Half medium changes with cNEM (1:1 advanced DMEM/F12: neurobasal medium, 1× neural induction supplement, Gibco) were performed every other day. Cultures were kept under constant orbital shaking (88 rpm).

After 4 weeks, neurospheroids were fixed with 4% PFA, and 10 μm thick slices were made for staining. The slices were permeabilized using 0.1% Triton-X-100 in TBS for 30 min before blocking the sections for 1 h with 20% goat serum in TBS. The primary antibody (mouse anti-Tuj1, 1:250, MAB1195, R&D Systems) was diluted in 10% milk in TBS and was applied overnight at 4 °C. Following washing, secondary antibody (goat anti-mouse IgG AF555, 1:200, A21425, Invitrogen) incubation was done for 1 h at RT. Counterstaining with DAPI was performed for 10 min. Pictures were taken using a BX51 fluorescence microscope equipped with an Olympus DP71 digital camera (Olympus).

### Cell viability assays

In the neuroblastoma monoculture, cell death was induced via incubation with different SNAP concentrations (*S*-nitroso-*N*-acetylpenicillamine, 100–800 μM, Abcam). Cells were either pre-treated for 24 h or co-treated with various rIL-13 concentrations (5, 33.3, 50, and 500 ng/ml). After 48 h or 72 h, 0.5 mg/ml MTT (3[4,5-Dimethylthiazol-2-yl]-2,5-diphenyltetrazolium bromide, Sigma-Aldrich) was added to the standard medium for 4 h, after which the cells were lysed and the formazan crystals dissolved in a mixture of DMSO and glycine (0.1 M). Absorbance was measured at a wavelength of 570 nm.

In the brain slices culture, cell death was induced via incubation with 100 μM NMDA (*N*-methyl-d-aspartic acid, Sigma-Aldrich) for 4 h. After washing, PI (propidium iodide, 5 µg/ml, Invitrogen) was added for 10 min. Pictures were taken using a Nikon Eclipse 80i fluorescence microscope. The PI uptake (fluorescence intensity) from the cells of the cornu ammonis region (CA1–CA3) of the hippocampus was analyzed using ImageJ software.

Neurospheroids (4 weeks) were subjected to control or oxygen–glucose deprivation (OGD) conditions for 6 h. OGD conditions were achieved by incubating the neurospheres in glucose-free NSC medium (DMEM/F12 w/o l-glutamin, w/o HEPES, w/o glucose [VWR], 1× B27 [Gibco], rhEGF and rhFGF-2 [20 ng/ml, Immunotools]) in a humidified Bactron IV anaerobic chamber (Shel Lab, USA) containing 0% O_2_, 5% CO_2_, 95% N_2_. After OGD, glucose (4.5 mg/ml, Gibco) was administered, and spheroids were brought back to atmospheric O_2_ level (21%). After 90 min of reoxygenation, human rIL-13 (10 ng/ml, Immunotools) was added to the medium for 48 h. Luminescence of the neurospheroids was determined 96 h following reoxygenation by incubating with 150 µg/ml Beetle luciferin (Promega) for 48 h.

### Statistics

Data analysis was performed using GraphPad Prism version 7 (GraphPad Software). D’Agostino and Pearson omnibus test was used to assess normality. The BMS results were analyzed using a two-way ANOVA for repeated measurements with a Bonferroni post hoc test. GFAP and Iba-1 intensity were analyzed using a two-way ANOVA with a Bonferroni post hoc test. All other data were analyzed using either a one-way ANOVA with a Bonferroni post hoc test or with a non-parametric Kruskal Wallis test with a Dunn’s multiple comparison test. Data are presented as mean ± standard error of the mean (SEM). Differences with *P* values < 0.05 were considered significant (*P < 0.05, **P < 0.01, ***P < 0.001, and ****P < 0.0001).

## Results

### IL-13 secreting Mφs express receptors for lesion-associated chemokines and have an anti-inflammatory phenotype

**Fig. 1:**
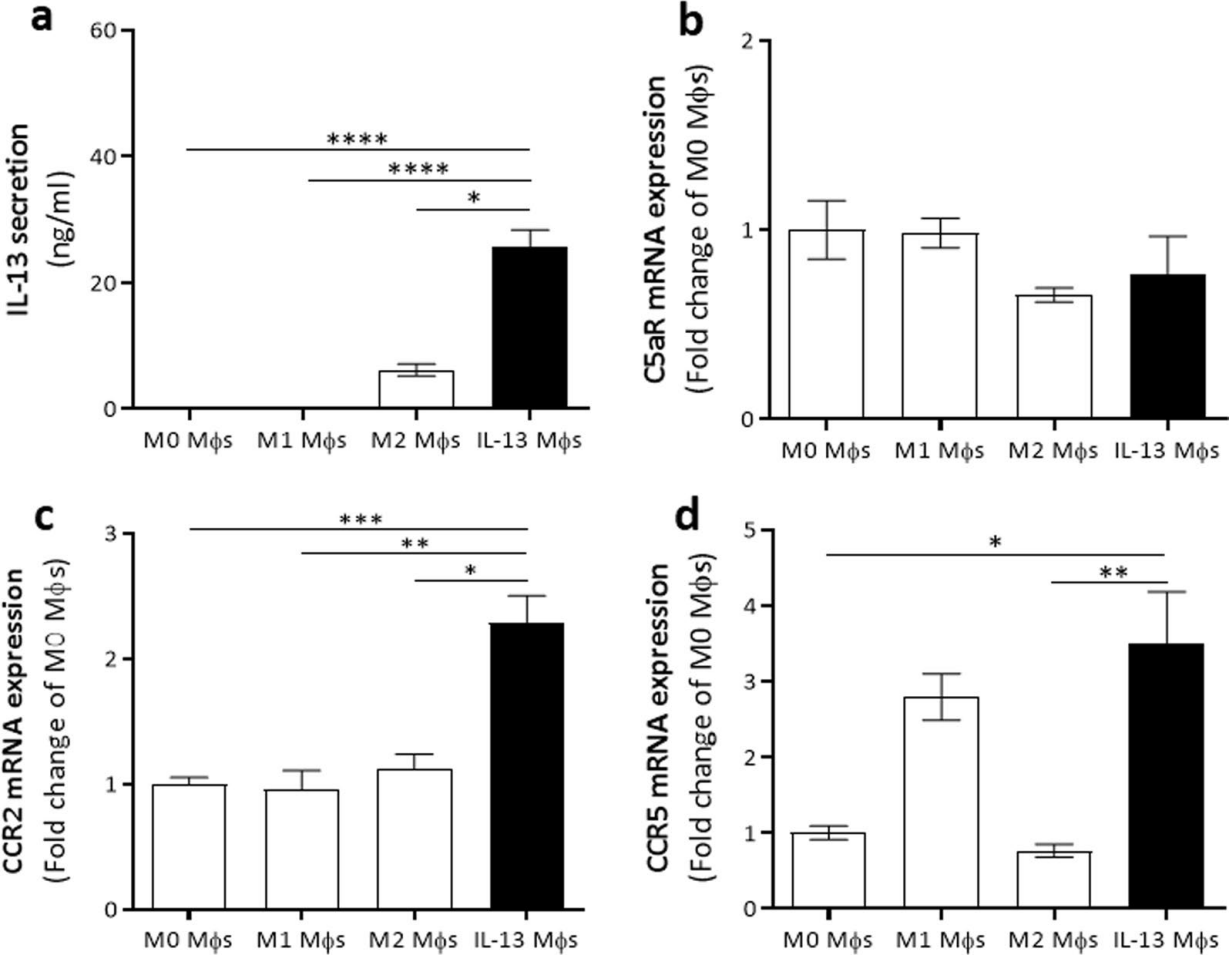
IL-13 secreting Mφs show upregulation of CCR2 and CCR5 gene expression but not C5aR. **a–d** Mφs were isolated from C57BL/6J mice. **a** IL-13 secretion by IL-13 Mφs was significantly higher than M0, M1, or M2 Mφs as determined by an ELISA. *n* = 9–13. **b**–**d** qPCR analyses showed that the gene expression of C5aR (**b**) was not changed, whereas IL-13 Mφs showed significant upregulation of CCR2 (**c**) and CCR5 (**d**) expression compared to both M0 and M2 Mφs. Data were normalized to M0 Mφs and represent mean ± SEM. *n* = 4–7. Kruskal–Wallis test with Dunn’s correction.*P < 0.05, **P < 0.01, ***P < 0.001, and ****P < 0.0001

Mφs are reported to migrate towards inflammatory sites after CNS trauma [16]. Therefore, they were selected as carriers of IL-13 to provide a continuous and stable secretion of this anti-inflammatory cytokine locally at the lesion site. IL-13 secretion by IL-13 Mφs, generated by molecular cloning, was confirmed via ELISA. IL-13 Mφs secreted significantly more IL-13 compared to M0, M1 (LPS-stimulated), and M2 (rIL-13-stimulated) Mφs (Fig. 1a). To address the homing potential of IL-13 Mφs, the gene expression of different receptors for lesion-associated chemokines was analyzed [25, 26]. No upregulation of C5aR was observed, whereas IL-13 Mφs had increased CCR2 (C–C chemokine receptor 2) and CCR5 gene expression compared to M0 Mφs (Fig. 1b–d). Notably, the expression of these receptors by IL-13 Mφs was also significantly higher compared to M2 Mφs (Fig. 1c, d).

**Fig. 2:**
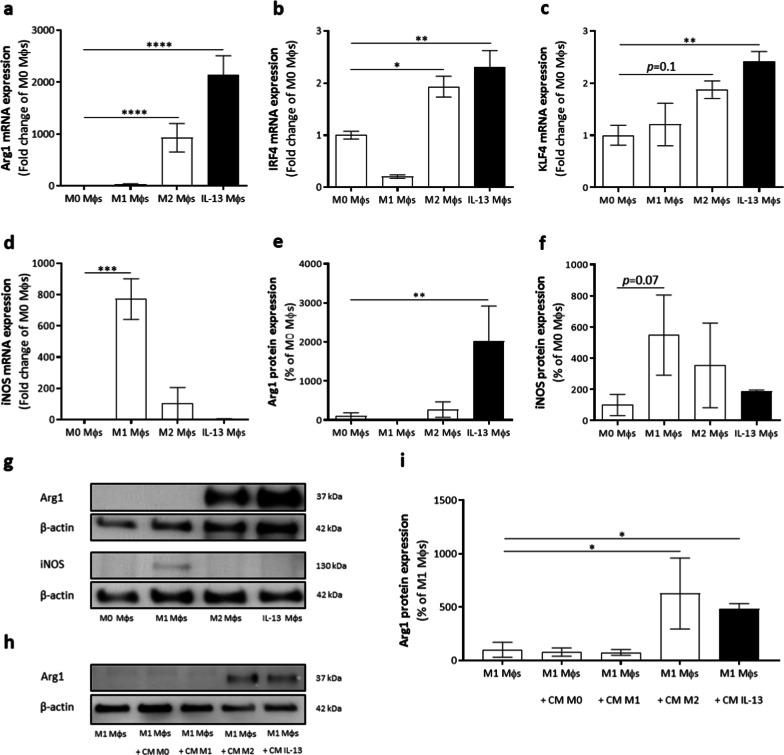
IL-13 Mφs have an anti-inflammatory phenotype and secrete functional IL-13. **a–i** Mφs were isolated from C57BL/6J mice. **a**–**d** Gene expression analysis by qPCR showed that IL-13 Mφs have an upregulation of Arg1 (**a**) and the conserved genes IRF4 (**b**) and KLF4 (**c**), while no increased expression of iNOS (**d**) was observed relative to M0 Mφs. Data were normalized to M0 Mφs and represent mean ± SEM. *n* = 8–14. **e**, **f** Western blot quantification showed increased expression of Arg1 (**e**) but no upregulation of iNOS (**f**) by IL-13 Mφs compared to M0 Mφs. Data were normalized to M0 Mφs and represent mean ± SEM. *n* = 4–5. **g** Representative immunoblot of Arg1 and iNOS expression. **h**, **i** Representative image (**h**) and quantification (**i**) of the western blot analysis of Arg1 expression. Following incubation with the conditioned medium (CM) of IL-13 Mφs, M1 Mφs showed Arg1 protein expression. Data were normalized to M1 Mφs and represent mean ± SEM. *n* = 3–4. Kruskal–Wallis test with Dunn’s correction. *P < 0.05, **P < 0.01, ***P < 0.001, and ****P < 0.0001

Next, an extensive phenotypical analysis of typical anti- and pro-inflammatory markers expressed by the IL-13 Mφs was performed before in vivo transplantation. Gene data analysis showed that expression of Arg1, FIZZ1, and Ym1 was significantly increased in IL-13 Mφs, as well as in M2 Mφs, compared to M0 Mφs (Fig. 2a and Additional file 2: Fig. S1a, b). Notably, IL-13 Mφs also showed an increased expression of FIZZ1 and Ym1 compared to M2 Mφs (Additional file 2: Fig. S1a, b). No significant upregulation of CD206 expression by IL-13 Mφs was detected (Additional file 2: Fig. S1c). In addition, the gene expression of the conserved anti-inflammatory markers interferon regulatory factor 4 (IRF4) and Kruppel-like factor 4 (KLF4) was analyzed [27]. Both IL-13 Mφs and M2 Mφs showed an upregulation of these genes compared to M0 Mφs (Fig. 2b, c). In contrast to M1 Mφs, IL-13 Mφs showed no upregulation iNOS expression or other M1-related genes (tumor necrosis factor-alpha [TNF-α], CD38, and CD86) compared to M0 Mφs (Fig. 2d and Additional file 2: Fig. S1d–f). In line with these gene expression data, IL-13 Mφs showed a significant upregulation of Arg1 protein expression but no upregulation of iNOS (Fig. 2e–g). To verify that the IL-13 secreted by IL-13 Mφs was functional, we investigated whether CM of these cells could induce Arg1 expression in M1 Mφs, as the majority of perilesional Mφs tend to display a pro-inflammatory phenotype in vivo [28]. Incubation with CM of IL-13 Mφs or M2 Mφs was able to induce Arg1 expression by M1 Mφs (Fig. 2h, i).

### IL-13 Mφs maintain their IL-13 secretion and anti-inflammatory markers upon a pro-inflammatory stimulus in vitro

**Fig. 3:**
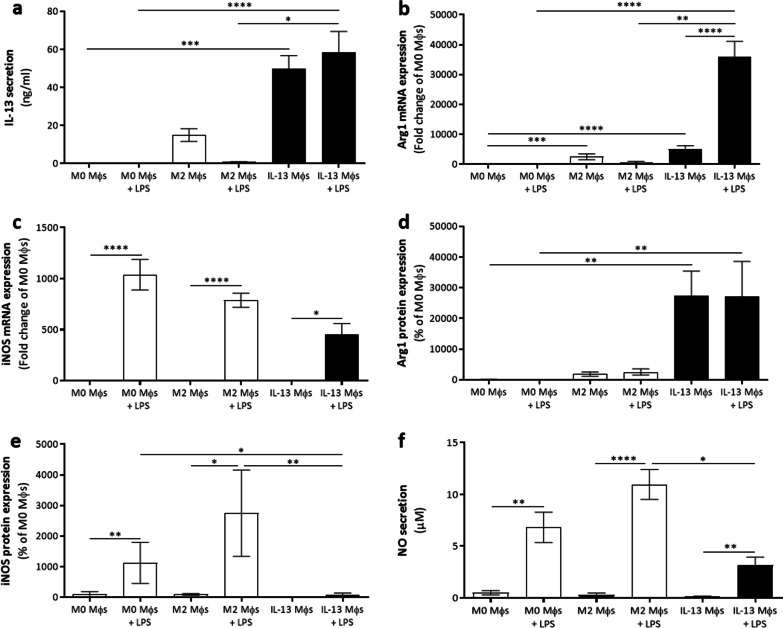
IL-13 Mφs maintain their IL-13 secretion and anti-inflammatory markers upon LPS stimulation. **a**–**f** Mφs were isolated from C57BL/6J mice. M0, M2, and IL-13 Mφs were left unstimulated (control) or were stimulated with LPS for 24 h. **a** IL-13 secretion, determined by ELISA, was not changed following LPS stimulation in IL-13 Mφs. Data represent mean ± SEM. *n* = 5–6. **b**, **c** Gene expression, determined by qPCR, showed that LPS exposure significantly increased Arg1 (**b**) and iNOS (**c**) expression by IL-13 Mφs compared to their untreated control. Data were normalized to M0 Mφs and represent mean ± SEM. *n* = 8–9. **d**, **e** Quantification of the western blot analysis showed that the protein expression of Arg1 (**d**) and iNOS (**e**) were not altered by LPS stimulation in IL-13 Mφs. Data were normalized to M0 Mφs and represent mean ± SEM. *n* = 4–6. **f** Using a Griess assay, NO was measured in the medium. Although NO secretion was significantly increased in the LPS-stimulated IL-13 Mφs compared to their untreated control, the secretion was significantly reduced compared to the M2 Mφs stimulated with LPS. Data represent mean ± SEM. *n* = 9. Kruskal–Wallis test with Dunn’s correction. *P < 0.05, **P < 0.01, ***P < 0.001, and ****P < 0.0001

Given the presence of a pro-inflammatory environment at the lesion site after SCI, we determined whether IL-13 Mφs could maintain their anti-inflammatory features when exposed to a pro-inflammatory stimulus in vitro. Interestingly, IL-13 secretion from IL-13 Mφs remained unaltered following LPS stimulation (Fig. 3a). Gene expression analyses showed that Arg1 expression significantly increased in IL-13 Mφs after LPS stimulation compared to their untreated control (Fig. 3b). While FIZZ1 level decreased considerably after LPS stimulation, Ym1 expression by IL-13 Mφs remained unaffected (Additional file 3: Fig. S2a, b). Notably, the expression of these anti-inflammatory markers by IL-13 Mφs was still significantly upregulated compared to LPS-stimulated M0 and M2 Mφs (Additional file 3: Fig. S2a, b). CD206 gene expression was significantly reduced in the LPS-stimulated IL-13 Mφs compared to the untreated control, although no upregulation was observed in IL-13 Mφs compared to M0 Mφs (Additional file 2: Fig. S1c and Additional file 3: Fig. S2c). Concerning the pro-inflammatory markers, iNOS expression was induced in IL-13 Mφs after LPS stimulation compared to their unstimulated condition (Fig. 3c). This was similar to M0 and M2 Mφs, which also displayed increased iNOS expression compared to their controls (Fig. 3c). Similar results were obtained for TNF-α, CD38, and CD86 (Additional file 3: Fig. S2d–f). In contrast to this gene expression data, protein expression analysis showed that Arg1 and iNOS expression remained unaltered in IL-13 Mφs following LPS stimulation, whereas M0 and M2 Mφs displayed an increased iNOS protein level after LPS incubation compared to their untreated controls (Fig. 3d, e). In addition, nitric oxide (NO) secretion was measured to analyze iNOS activity. Upon LPS stimulation, IL-13 Mφs produced more NO compared to their untreated controls (Fig. 3f). Notably, this was significantly decreased compared to the NO secreted by M2 Mφs stimulated with LPS (Fig. 3f).

### Transplantation of IL-13 Mφs improves functional and histopathological outcome after spinal cord injury

**Fig. 4:**
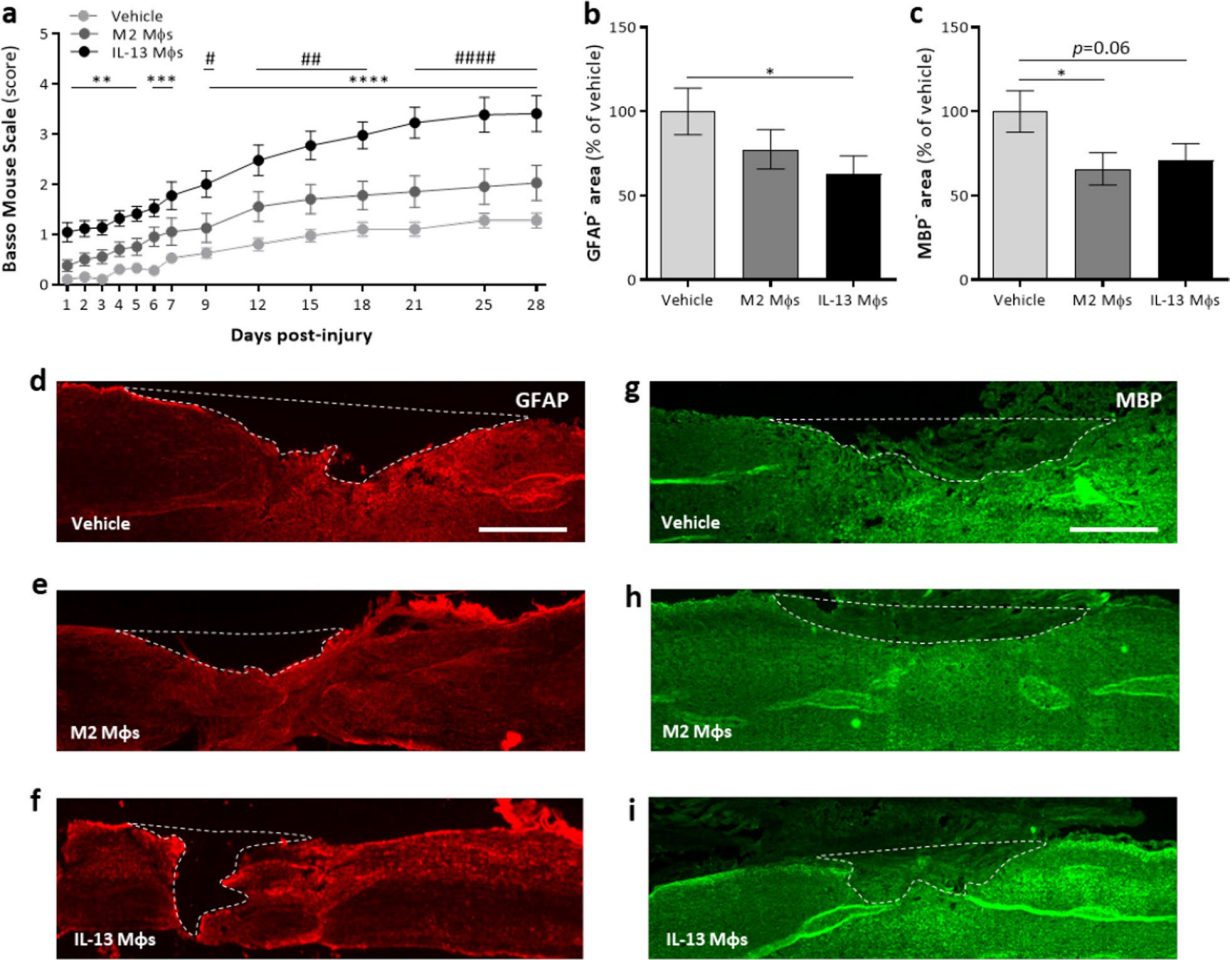
Transplantation of IL-13 Mφs improves functional and histopathological outcomes after spinal cord injury. **a**–**i** Immediately following injury, C57BL/6j mice received vehicle, M2 Mφs or IL-13 Mφs. **a** IL-13 Mφs transplantation significantly improved functional outcome compared to vehicle and M2 Mφs. Data are shown as mean ± SEM. *n* = 20–22 mice/group.*IL-13 Mφs versus vehicle, # IL-13 Mφs versus M2 Mφs. Two independent experiments. **b**, **c** Quantification of lesion size (**b**) and demyelinated area (**c**), determined by the GFAP and MBP negative area, showed that these were reduced in IL-13 Mφ-treated mice compared to control. Data were normalized to vehicle and are shown as mean ± SEM. *n* = 11–13 mice/group. **d**–**i** Representative images from the spinal cord sections are shown. Lesion size (GFAP^−^, **d**–**f**) and demyelinated are (MBP^−^, **g**–**i**) were determined as depicted by the dotted white line. Scale bar = 500 µm. One way ANOVA (**b**, **c**) or two-way ANOVA (**a**) with Bonferroni post hoc test. *P < 0.05, **P < 0.01, and ****P < 0.0001

To investigate the therapeutic potential of IL-13 Mφs following SCI, mice received an intraspinal injection with either vehicle or IL-13 Mφs immediately after injury induction in the perilesional area. As the in vitro results showed that M2 Mφs adopt a mixed phenotype upon a pro-inflammatory stimulus, these cells were included to test whether stable IL-13 secretion and anti-inflammatory gene expression are prerequisites for beneficial effects. Functional recovery was measured for 4 weeks using the BMS score. Mice treated with IL-13 Mφs showed significantly improved functional recovery compared to vehicle and M2 Mφ groups (Fig. 4a). Accordingly, histological analyses indicated significantly reduced lesion size and a trend towards decreased demyelination in IL-13 Mφ-treated mice compared to vehicle control (Fig. 4b–i). M2 Mφ treatment did not affect functional outcome, although a significant decrease in demyelinated area compared to vehicle was shown (Fig. 4a, c, g–i). No differences in astrogliosis were observed between the different groups (Additional file 4: Fig. S3a, c–e). To evaluate cell survival, GFP-labelled M2 and IL-13 Mφs were injected into the spinal cord. Noteworthy, the IL-13 Mφs were not detected in the spinal cord at 8 dpi in contrast to M2 Mφs (Additional file 5: Fig. S4a–d).

### Transplantation of IL-13 Mφs induces an anti-inflammatory shift in phagocytes at the lesion site

**Fig. 5:**
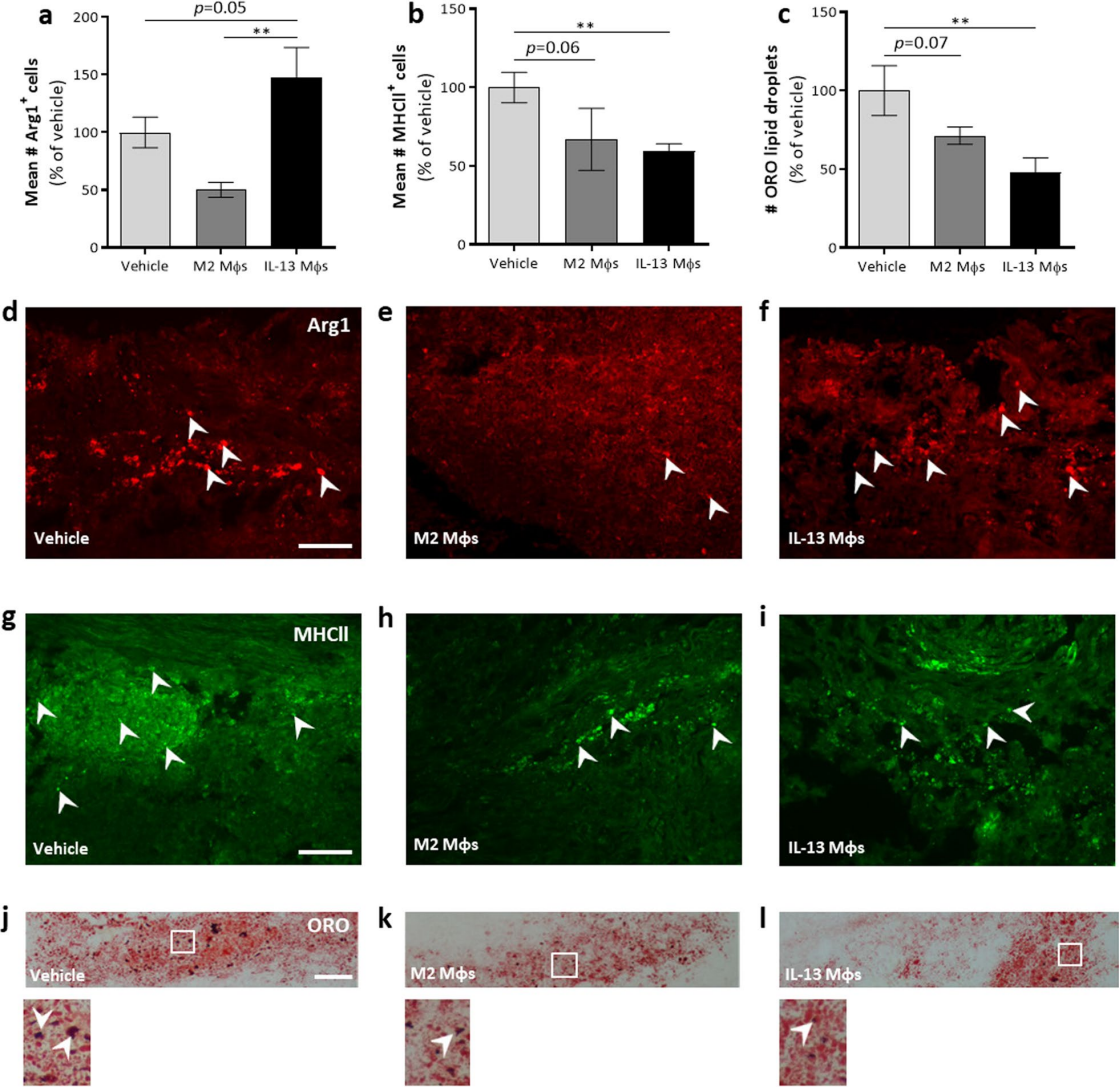
Transplantation of IL-13 Mφs induces a more anti-inflammatory environment at the lesion site. **a**–**l** Immediately following injury, C57BL/6j mice received vehicle, M2 Mφs or IL-13 Mφs. **a**, **b** Number of Arg1^+^ (**a**) at the lesion site was increased, while the amount of MHCII^+^ (**b**) cells was significantly decreased in IL-13 Mφ-treated mice compared to vehicle. Data were normalized to vehicle and are shown as mean ± SEM. *n* = 4–7 mice/group. **c** The number of ORO^+^ lipid droplets at the lesion site was significantly reduced in IL-13 Mφ-treated mice compared to vehicle. Data were normalized to vehicle and are shown as mean ± SEM. *n* = 9 mice/group. **d**–**l** Representative images of the Arg1 (**d**–**f**), MHCII (**g**–**i**), and ORO (**j**–**l**) staining at the lesion. Arg1^+^ or MHCll^+^ cells, as well as ORO droplets, are indicated by white arrows. Scale bar = 100 µm. Kruskal–Wallis test with Dunn’s correction (**a**, **b**) or one-way ANOVA with Bonferroni post hoc test (**c**). **P < 0.01

As IL-13 is one of the main inducers of an alternatively activated Mφ/microglia phenotype, we assessed the phenotype of perilesional Mφs/microglia to investigate whether IL-13 Mφ transplantation induced an anti-inflammatory shift. First, we showed that cell transfer did not affect the presence of Mφs/microglia at the lesion site (Additional file 4: Fig. S3b, f–h and Additional file 6: Fig. S5a). Second, the number of alternatively (Arg1^+^) and classically (MHCII^+^) activated phagocytes at the lesion site was determined (Fig. 5a, b, d–i). IL-13 Mφ treatment increased the amount of Arg1^+^ cells at the lesion site while reducing the number of MHCII^+^ cells compared to vehicle (Fig. 5a, b, d–i). Besides MHCII expression, foamy Mɸs are considered to be detrimental inflammatory cells in SCI [29]. Therefore, we determined the perilesional lipid load as an indirect measurement for the presence of foamy cells. Mice treated with the IL-13 Mφs had a significantly reduced number of lipid droplets versus vehicle (Fig. 5c, j–l).

### Transplantation of IL-13 Mφs skews the lesion site towards a neuroprotective environment

**Fig. 6:**
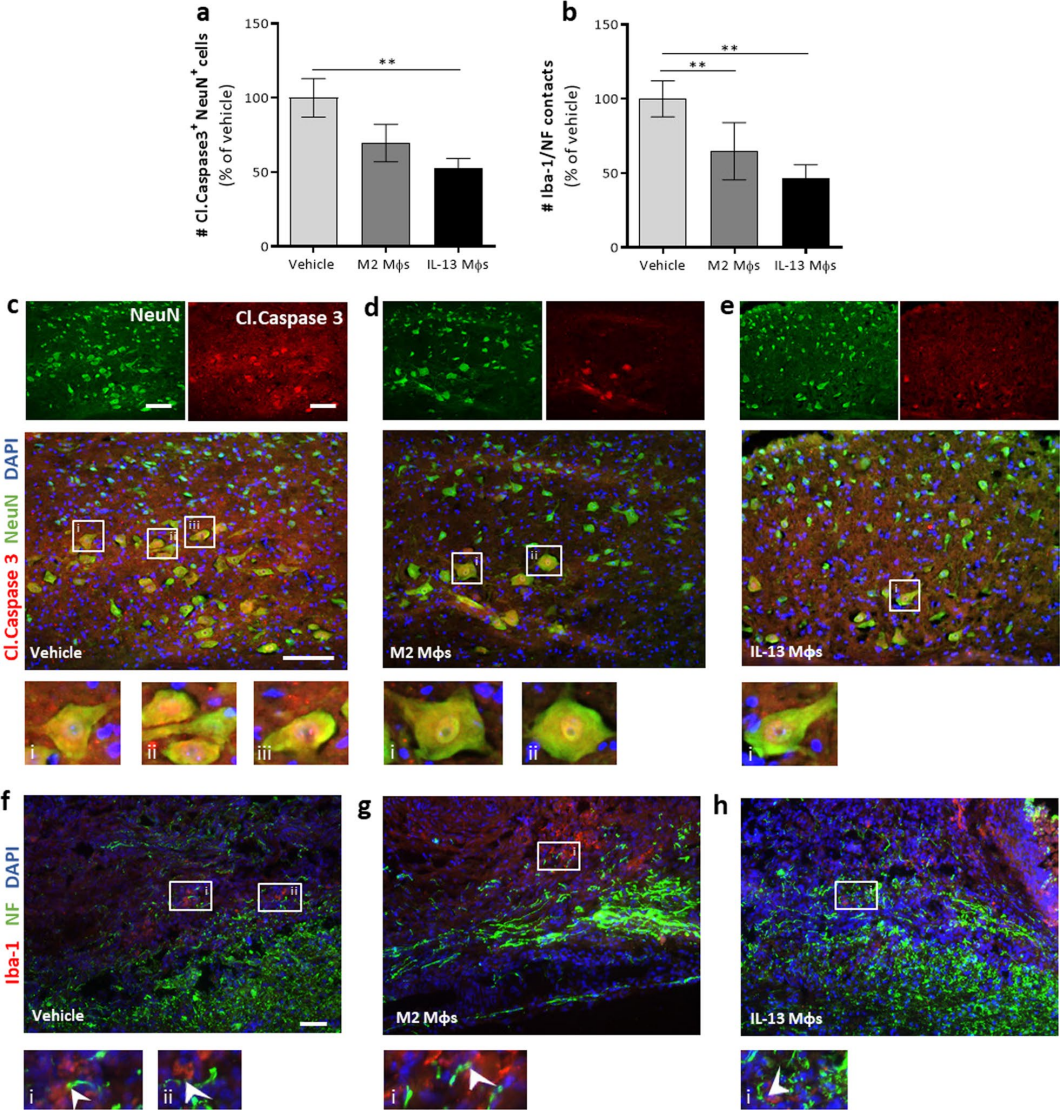
Transplantation of IL-13 Mφs induces a neuroprotective environment at the lesion site. **a**–**h** Immediately following injury, C57BL/6j mice received vehicle, M2 Mφs or IL-13 Mφs. **a** Quantification of the number cleaved (Cl.) caspase 3^+^NeuN^+^ neurons at the lesion site showed reduced neuronal cell death in the IL-13 Mφ group compared to vehicle. Data were normalized to vehicle and are shown as mean ± SEM. *n* = 3–8 mice/group. **b** Number of Mφs/microglia (Iba-1) and dystrophic axon (NF) contacts was significantly reduced in the IL-13 Mφ group compared to vehicle. Data were normalized to vehicle and are shown as mean ± SEM. *n* = 9–13 mice/group. **c**–**e** Representative images of the Cl. caspase 3-NeuN staining at the lesion site. Single stainings are shown above the merged image. Examples of double-positive cells are indicated by the white boxed regions (i–iii) and are shown at a higher magnification underneath. Scale bar = 100 µm. **f**–**h** Overview images of the areas from the lesion site used for quantification. The white boxed regions (i–iii) in images (**f**–**h**) are shown at a higher magnification underneath. The white arrows indicate examples of Mφs/microglia and dystrophic axon contacts. Scale bar = 50 µm. Kruskal–Wallis test with Dunn’s correction (**a**) or one-way ANOVA with Bonferroni post hoc test (**b**). **P < 0.01

The abovementioned results indicate that IL-13 Mφs transplantation enforced an anti-inflammatory perilesional milieu. Next, we investigated whether this change in environment also provided neuroprotection. First, the neuroprotective effect of IL-13 Mφs was determined through double staining for cleaved caspase 3 and NeuN to detect apoptotic neurons. Mice treated with IL-13 Mφs had reduced neuronal cell death compared to vehicle (Fig. 6a, c, e). Furthermore, the M2 Mφs group only displayed a non-significant trend towards reduced neuronal cell death relative to control (Fig. 6a, c, d). Second, the impact of IL-13 Mφ transplantation on the detrimental phagocyte–axon contacts was investigated. This was determined via quantification of the contacts between Mφs/microglia (Iba-1^+^ cells) and dystrophic axons (NF^+^ cells showing end bulbs) at the lesion site [4]. IL-13 Mφs significantly reduced the number of detrimental contacts compared to vehicle control (Fig. 6b, f, h). A similar effect was observed in the mice treated with the M2 Mφs (Fig. 6b, f, g).

### IL-13 is critical for the beneficial effects of IL-13 Mφs after spinal cord injury

**Fig. 7:**
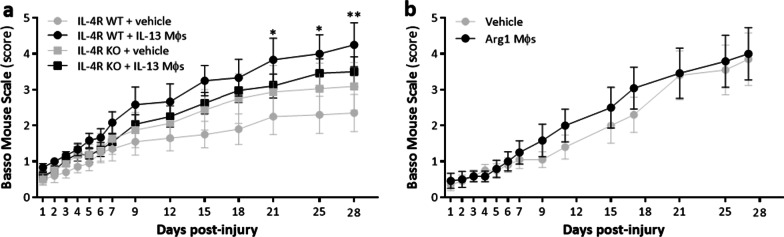
IL-13 is the key factor behind the beneficial effects of IL-13 Mφs in vivo. **a** IL-4Rα WT or KO BALB/c mice received vehicle or IL-13 Mφs immediately following injury. Functional recovery was improved by transplantation of IL-13 Mφs in WT mice but not in IL-4Rα KO mice. Data are shown as mean ± SEM. *n* = 6–12 mice/group. *WT + IL-13 Mφs versus vehicle. Two independent experiments. **b** C57BL/6J mice received vehicle or Arg-1 Mφs immediately following injury. No difference in functional recovery was observed between experimental groups. Data are shown as mean ± SEM. *n* = 10–12 mice/group. One independent experiment. Two-way ANOVA with Bonferroni post hoc test. *P < 0.05, **P < 0.01

We have shown that IL-13 Mφs have anti-inflammatory and neuroprotective effects in vivo, accompanied by improved functional recovery after SCI*.* This can be attributed to either IL-13 secretion or the anti-inflammatory phenotype of the IL-13 Mφs (e.g., Arg1 expression). To investigate this, we used two different approaches. First, IL-13 Mφs were administered to IL-4Rα KO mice. These mice lack not only IL-4 signaling but also IL-13 signaling as IL-13Rα1 activation requires heterodimerization with the IL-4Rα, which is prevented in the IL-4Rα KO model [30]. After SCI induction and cell transplantation, functional recovery was analyzed for 4 weeks using the BMS score. IL-13 secretion by IL-13 Mφs was confirmed by ELISA (Additional file 7: Fig. S6a). WT control and vehicle administration were included as a control for KO-induced effects and to ensure positive outcome after IL-13 Mφ transplantation, respectively. The IL4Rα KO did not affect functional recovery (Fig. 7a, IL-4Rα KO mice + vehicle versus WT controls + vehicle). Transplantation of IL-13 Mφs into the spinal cord of IL-4Rα KO mice did not affect functional recovery (Fig. 7a, IL-4Rα KO mice + IL-13 Mφs versus IL-4Rα KO + vehicle), whereas transplantation of IL-13 Mφs improved outcome in WT controls compared to vehicle treatment (Fig. 7a, WT + IL-13 Mφs versus WT + vehicle). Second, Arg1 Mφs were transplanted, which represent Mφs that do not secrete IL-13 but overexpress the typical anti-inflammatory Mφ/microglia marker Arg1 (Additional file 7: Fig. S6b, c). Again, functional recovery was analyzed. Transplantation of Arg1 Mφs did not improve functional recovery compared to vehicle control mice (Fig. 7b).

**Fig. 8:**
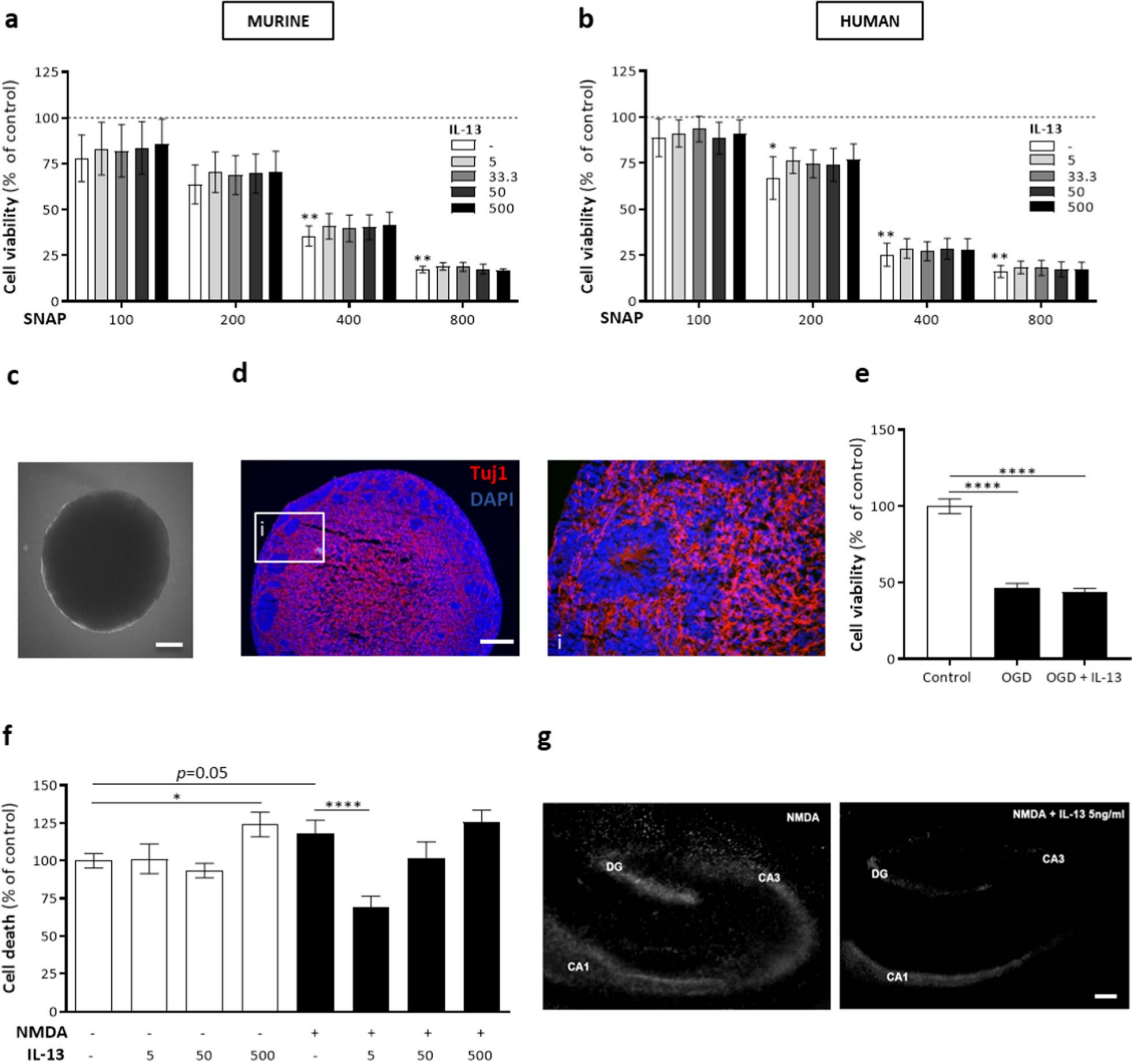
IL-13 provides indirect neuroprotection. **a**, **b** Neuro2A (**a**) or SH-SY5Y (**b**) cells were treated for 72 h with different concentrations of SNAP (µM) with or without IL-13 (ng/ml). IL-13 did not protect against cell death determined by an MTT assay. Data were normalized to untreated control (= dotted black line) and are shown as mean ± SEM. *n* = 4–5. **c**, **d** Representative images of 4-week old neurospheroids obtained from luciferase-expressing hiPSC-NSCs. **c** Brightfield image of neurosphere. Scale bar = 500 µm. **d** Immunofluorescent image of neurospheroid showing strong Tuj1 expression. White box region (i) is shown at a higher magnification. Scale bar = 200 µm. **e** Neurospheroids from luciferase-expressing hiPSC-NSCs were subjected to hypoxic conditions (OGD) for 6 h and were left untreated or were incubated for 48 h with IL-13. No protective effect of IL-13 on cell viability was observed. Data were normalized to untreated control and are shown as mean ± SEM. *n* = 14–15. **f** Hippocampal–entorhinal brain slices derived from BALB/c mice were incubated for 72 h with IL-13 (ng/ml) with or without NMDA (100 µM). A low concentration of IL-13 (5 ng/ml) significantly reduced cell death measured via a PI staining. However, even without NMDA administration, a high dose of IL-13 (500 ng/ml) induced cell death. Data were normalized to untreated control and are shown as mean ± SEM. *n* = 17–38 slices/condition. **g** Representative images of the PI assay, depicting the hippocampal cornu ammonis (CA) area 1 and 3, showing a decrease in fluorescence intensity when slices were treated with NMDA + IL-13 (5 ng/ml) compared to NMDA treatment alone. *DG* dentate gyrus. Scale bar = 250 µm. Kruskal–Wallis test with Dunn’s correction (**a**, **b**) or one-way ANOVA with Bonferroni post hoc test (**e**, **f**). *P < 0.05, **P < 0.01, and ***P < 0.001

These results indicate that IL-13 is critical for functional recovery and neuroprotective effects induced by IL-13 Mφs, which are absent in mice deficient for IL-4Rα signaling. Next, we investigated in vitro whether IL-13 was directly responsible for neuroprotection. We used murine and human neuroblastoma cell lines and induced cell death via NO, which plays a vital role in secondary injury neurotoxicity [31]. Of note, we confirmed the presence of the IL-13Rα1 and IL-4Rα on these cells (Additional file 8: Fig. S7a, b). Overall, treatment with different concentrations of IL-13 did not affect cell viability (Additional file 8: Fig. S7c, d). Incubation (72 h) of these cells with varying concentrations of SNAP (100–800 µM), an NO donor, significantly induced cell death at high doses (75% at 800 µM, Fig. 8a, b). No rescue effect was observed by incubation with IL-13 (5–500 ng/ml, Fig. 8a, b). Likewise, 48 h co-treatment or 24 h pretreatment with IL-13 did not affect cell viability (Additional file 8: Fig. S7e–h). In addition, we used a neurospheroid culture obtained from hiPSC-NSCs to analyze the direct effects of IL-13 in a more complex set-up which approaches the in vivo structural organization of cells. Four-week old spheroid cultures strongly expressed the neuronal marker Tuj1 (Fig. 8c, d). Using an OGD assay, we mimicked a state of hypoxia, which is also induced after SCI [31]. Following these experimental conditions, neuronal viability was reduced by 50% (Fig. 8e). However, IL-13 treatment did not influence cell viability (Fig. 8e).

The abovementioned results suggest that IL-13 does not have direct neuroprotective actions. Therefore, we used a murine brain slice model, which is a more complex in vitro trauma model to maintain the interaction between CNS neural and immune cells to include analyses of indirect effects induced by IL-13 on neurons. In this case, we used a model of NMDA-induced toxicity as NMDA contributes to secondary cell death via glutamate excitotoxicity [31]. In this model, NMDA treatment resulted in 18% cell death (Fig. 8f). A low concentration of IL-13 (5 ng/ml) protected against NMDA-induced cell death (Fig. 8f, g). Surprisingly, a high dose of IL-13 (500 ng/ml) was cytotoxic even in the absence of NMDA treatment (Fig. 8f).

## Discussion

It is generally accepted that neuroinflammation is a significant driver of secondary injury-related damage in SCI. During the last years, the potential of IL-13 as a treatment for neuroinflammation has been shown by us and others in different animal models of neurodegeneration [9, 12–15, 19, 32]. Here, we show for the first time that IL-13 delivery by genetically modified Mφs improves functional repair after SCI and that these beneficial effects are mediated by IL-13 and not the M2a phenotype of the IL-13 Mφs. Mφs were selected as carriers above MSCs due to their strong homing ability towards inflammatory sites and because MSC-mediated actions after SCI were attributed to the induction of M2a Mφs [15, 33]. In this study, we demonstrate that transplantation of IL-13 Mφs after SCI is an efficient approach for eliciting anti-inflammatory effects and thereby create a neuroprotective environment less prone to neuronal death and axonal retraction. Our study supports IL-13 administration as an effective immunomodulatory therapy and underlines the importance of reprogramming local Mφs/microglia to provide neuroprotection and improve functional outcome after SCI.

IL-13 secretion by Mφs has not been previously reported under physiological conditions, only in the context of pulmonary fibrosis [34]. Through genetic transfer, we induced IL-13 secretion by Mφs. Phenotyping of these IL-13 Mφs prior to in vivo administration showed that IL-13 Mφs secreted IL-13 at similar concentrations to that used for ex vivo polarization of M2a Mφs [12, 13]. In addition, CCR2 and CCR5 upregulation by IL-13 Mφs suggests an increased homing potential towards the lesion site, as their ligands, CCL2, and CCL3 are upregulated during the 1st days after SCI [16, 26]. Whether the upregulation of these factors in the IL-13 Mφ group also contributes to neuroprotection remains to be elucidated. The effects of the CCL2–CCR2 pathway after SCI are controversial. While CCR2 deficiency improves outcome, CCL2 contributes to M2 Mφ polarization [26, 35]. Interestingly, CCL2 treatment counteracted NMDA-induced cell death of motor neurons [36, 37]. The CCR5 axis has been described to play a pro-inflammatory role after SCI, as in the absence of CCR5 or its ligand, CCL3, recovery was improved [38, 39]. Therefore, it was rather surprising that CCR5 was upregulated by IL-13 Mφs. However, the role of CCR5 in SCI seems to be more complex as recombinant CCL5 treatment caused upregulation of M2 Mφ and axonal regeneration markers [26]. Overall, CCL2 and CCL5 have been described to be able to provide anti-inflammatory actions [26, 35]. Yet, since recombinant IL-13 alone has a similar neuroprotective effect in the brain slice culture, it is feasible that the CCR2 and CCR5 upregulation in IL-13 Mφs plays a minor role in protecting the spinal cord after lesion. The polarization of the IL-13 Mφs was indicated by the upregulation of the primary M2a markers Arg1, FIZZ1, and Ym1. Furthermore, their anti-inflammatory phenotype was more pronounced than M2 Mφs, potentially due to continuous autocrine signaling provided by IL-13.

As the inflammatory imbalance following SCI impedes regenerative processes, increasing the fraction of the anti-inflammatory Mφ/microglia phenotype has become a major therapeutic strategy. Ma et al. demonstrated that intravenous injection of M2 Mφs improved functional recovery after SCI [40]. However, Kigerl et al. showed that 3 days after intraspinal injection of GFP^+^-M2 Mφs, only 20–40% maintained their polarization status [28]. This is not surprising given that lesion-associated factors favor the classically activated Mφ phenotype. Based on these results, it is tempting to speculate that preventing an M1 switch of grafted cells is a prerequisite for functional improvement. To overcome this pitfall, we used genetically manipulated Mφs to secrete IL-13. Even under a robust inflammatory stimulus, these cells maintained IL-13 secretion, and their anti-inflammatory marker expression (Arg1, FIZZ1, and Ym1 expression) was more pronounced during inflammation than rIL-13 stimulated M2 Mφs. A similar observation was made by Boehler et al., who showed that IL-10 transduction was more potent than rIL-10 stimulation to maintain an M2 polarization upon inflammation [41]. Importantly, in our study, only IL-13 Mφs improved functional recovery after SCI, whereas the M2 Mφs did not. It is important to note that this is only a partial and no full recovery. IL-13 Mφ-treated mice reached an average score of 3.4, which is more than 2 points higher than the vehicle group. We consider this difference as biologically and therapeutically relevant, because it means that highly impaired mice which show only slight ankle movement develop a stepping pattern after IL-13 Mφ transplantation [20]. However, these data have to be interpreted with care, because in this study, only female mice have been used, and there is evidence that sex differences may influence the BMS outcome or the inflammatory response after SCI [42–44]. For example, female mice have a reduced lesion size and a higher Mφ number compared to males [42, 43]. Surprisingly, IL-13 Mφs did show an upregulation of iNOS mRNA but not of its protein expression after LPS stimulation. The link between IL-13 and iNOS gene expression was already observed in our previous studies [32, 45]. For example, IL-13 MSC treatment induced upregulation of Arg1 and iNOS mRNA levels in a cuprizone model [32]. Still, IL-13 delivery was associated with anti-inflammatory effects and suppressed demyelination.

The M1:M2 ratio is a crucial determinant of functional recovery after SCI [1]. As IL-13 is well-known for inducing alternative activation of phagocytes, we analyzed whether IL-13 Mφ injection caused a polarization shift in vivo. The perilesional environment of IL-13 Mφ-treated mice was converted as shown by an increased anti-inflammatory cell population (Arg1^+^ cells) and a decreased pro-inflammatory cell number (MHCII^+^ cells) without affecting the global Mφs/microglia cell presence. Notably, the increase in Arg1^+^ cells was absent in the mice who received M2 Mφs. This immunomodulation of local Mφs/microglia by IL-13 is in line with previous stroke and TBI reports [12–14]. Consistently, we previously showed in a study using IL-13 MSC that the Arg1-expressing population consisted mainly of invading monocytes/Mφs [15].

Lesional lipid load was analyzed as an indirect measurement of foamy cells, which are detrimental in SCI [46]. Mφs phagocytose apoptotic cells and myelin debris to pave the way for regeneration [47]. However, in SCI, excessive uptake overwhelms the intracellular lipid homeostasis, subsequently transforming them into large foamy, pro-inflammatory cells [46]. Wang et al. showed that Arg1 expression was reduced 7 dpi, while foam cell formation was observed [47]. As our results indicate an increase in Arg1^+^ cells, it may be expected that foam cell formation is reduced. Indeed, the ORO staining results showed an overall reduction in lipid load at the lesion site following IL-13 Mφ transplantation. Even though the underlying mechanism remains to be resolved, several studies indicate a role of IL-13 in phagocytosis. The importance of IL-13 in stimulating efferocytosis was described in a TBI animal model where repeated intranasal rIL-13 treatment increased the NeuN^+^Iba-1^+^ area [13]. In stroke, the STAT6/Arg1 pathway was found to induce efferocytosis by Mφs/microglia [48]. Mechanistically, Proto et al. revealed that IL-13 secretion activated IL-10 production by Mφs, which enhanced efferocytosis via autocrine signaling [49]. In addition, in a mouse model of atherosclerosis, IL-13 acted anti-atherogenic by inducing M2 polarization. In vitro, Mφs stimulated with IL-13 increased the uptake of oxLDL by CD36 upregulation and induced the expression of the lipid exporter (ABCA1), preventing foam cell formation, which is a major driver of atherosclerosis [50]. Based on these data, it is plausible that enhanced phagocytosis and/or processing may explain a reduction in foamy cells in our model.

Notably, the beneficial immunomodulatory effects of IL-13 Mφs were accompanied by neuroprotection. In animal models of TBI and stroke, IL-13 treatment also facilitated anti-inflammatory effects and reduced neuronal cell loss [12, 13]. Likewise, we observed a significant decrease in the number of cleaved caspase 3^+^ neurons in IL-13 Mφ-treated mice. Furthermore, damaged axons retract from the injury site after SCI, a process called axonal dieback [46, 51]. The significant contribution of Mφs to this phenomenon has been shown by Horn et al., who demonstrated a substantial reduction in axonal dieback following peripheral Mφ depletion [4]. In addition, M1 Mφs, in particular, have been reported to induce axonal retraction via destructive physical contacts [4]. IL-13 Mφ treatment did not affect the number of lesional Iba-1^+^ cells compared to vehicle. However, it reduced the number of contacts between dystrophic axons and phagocytes. Since the number of Iba-1^+^ cells was not influenced, this suggests that axonal dieback, a limiting factor for regeneration, was reduced [52]. Therefore, it can be speculated that induction of an anti-inflammatory perilesional environment by IL-13 Mφs creates a permissive milieu for axon regeneration by reducing neuronal cell death and axonal dieback. It cannot be excluded that direct effects on axonal plasticity may contribute to the observed beneficial effects. However, Vogelaar et al. have shown that there were no direct effects of IL-13 on axonal outgrowth in vitro [53].

To analyze whether the beneficial effects of IL-13 Mφs were either dependent on IL-13 itself or their altered M2a phenotype, we used an IL-4R*α* KO mouse model. IL-13 binding to its receptor, IL-13Rα1, will evoke the dimerization with the IL-4Rα for downstream pathway activation [54]. Deletion of this part will prevent IL-13-induced signaling. Noteworthy, IL-4 also exerts its effects through this receptor complex and IL-4 signaling will be lost as well [54]. However, following SCI, IL-4 levels are undetectable, challenging a significant role in the pathology [55]. In addition, we demonstrate that IL-13 Mφs do not secrete detectable levels of IL-4 (below detection range, Additional file 9: Table S2). Vehicle-treated WT and IL-4R*α* KO mice did not show differences in functional recovery after SCI. This is in line with Fenn et al., who did not observe reduced recovery after SCI in adult IL-4R*α* KO mice [56]. The functional improvement induced by IL-13 Mφs was abrogated in IL-4R*α* KO mice, highlighting the role of proper IL-4R*α* signaling. Besides their IL-13 secretion, IL-13 Mφs represent anti-inflammatory Mφs which overexpress Arg1 compared to rIL-13-stimulated M2 Mφs. To investigate whether this polarization was involved in their beneficial effects, Arg1 overexpressing Mφs, which do not secrete IL-13, were also grafted following SCI. However, no significant impact on functional recovery was observed. These results point towards IL-13 signaling as the critical mechanism by which IL-13 Mφs induce immunomodulation, neuroprotection, and functional recovery after SCI. Consistently, IL-13 delivery by MSCs was able to modulate Mφ/microglia polarization during the active inflammation phase in a cuprizone model [14]. As we were unable to trace the IL-13 Mφs 8 days after SCI, initial IL-13 secretion seems to be crucial for the functional recovery, while persistence of the cells at the lesion site appears not to be required.

Finally, our results indicate that the neuroprotective effect by IL-13 Mφs was not mediated by direct action of IL-13 on neurons. In both murine and human neuroblastoma cell lines, neither pre- nor co-treatment with IL-13 protected against NO-induced cell death. Similarly, no protective effect of IL-13 on the cell viability of OGD-subjected neurospheroids was observed. However, in an murine organotypic brain slice model, IL-13 (5 ng/ml) rescued neurons from NMDA-induced cell death. This suggests that IL-13 indirectly affects the survival of neurons via IL-13R-expressing microglia, which are present in organotypic brain slice culture but not in the neuronal monocultures. Consistent with these findings, Shin et al. reported that the induction of IL-13 in microglia contributed to the survival of neurons upon inflammation [57]. Accordingly, Miao et al. showed that IL-13 reduced neuronal death in an Mɸ neuronal co-culture by suppressing NO secretion, again highlighting the indirect neuroprotective effect of IL-13 [13]. Aside from the impact of IL-13 on Mφs/microglia, astrocytes can also be influenced. Stimulation of astrocytes with IL-13 induced brain-derived neurotrophic factor secretion, which reduces neuronal death, stimulates axonal regeneration and improves functional recovery following SCI [58, 59]. Regardless, we did not observe a significant difference in astrogliosis.

## Conclusions

To summarize, this study shows for the first time that perilesional transplantation of genetically modified Mφs that secrete IL-13 is a promising approach to improve functional recovery after SCI. Our data suggest that IL-13 elicits indirectly neuroprotection via stimulating anti-inflammatory responses in Mφs/microglia. In addition, IL-13 also possibly ameliorated axonal dieback facilitating regeneration. Our data strongly support the therapeutic use of IL-13 and consider it a prime candidate for effective immunomodulation after SCI to improve repair.

## Supplementary Information

**Additional file 1: Table S1.** Primers used for qPCR.

**Additional file 2: Figure S1.** IL-13 Mφs have an anti-inflammatory phenotype. **a**–**f** Mφs were isolated from C57BL/6J mice. qPCR showed that gene expression of the anti-inflammatory markers FIZZ1 (**a**) and Ym1 (**b**) were significantly increased in the IL-13 Mφs compared to M0 Mφs, whereas CD206 (**c**) was not. Pro-inflammatory gene expression of TNF-α (**d**), CD38 (**e**), and CD86 (**f**) were not induced in the IL-13 Mφs. Data were normalized to M0 Mφs and represent mean ± SEM. *n* = 12–14. Kruskal–Wallis test with Dunn’s correction. *P < 0.05, **P < 0.01, ***P < 0.001, and ****P < 0.0001.

**Additional file 3: Figure S2.** IL-13 Mφs maintain their anti-inflammatory markers upon LPS stimulation. **a**–**f** Mφs were isolated from C57BL/6J mice. M0, M2, and IL-13 Mφs were left unstimulated (control) or were stimulated with LPS for 24 h. **a**–**c** qPCR showed that upon incubation with LPS, IL-13 significantly decreased the expression of the anti-inflammatory markers FIZZ1 (**a**) and CD206 (**c**) expression, whereas their expression of Ym1 (**b**) was maintained. Data were normalized to M0 Mφs and represent mean ± SEM. *n* = 8–9. **d**–**f** Pro-inflammatory gene expression of TNF-α (**d**), CD38 (**e**), and CD86 (**f**) were induced in the IL-13 Mφs upon LPS incubation as determined by qPCR. Data were normalized to M0 Mφs and represent mean ± SEM. *n* = 8–9. Kruskal–Wallis test with Dunn’s correction (**b**, **d**, **e**) or one-way ANOVA with a Bonferroni post hoc test (**a**, **c**, **f**). *P < 0.05, **P < 0.01, ***P < 0.001, and ****P < 0.0001.

**Additional file 4: Figure S3.** IL-13 Mφ transplantation does not affect astrogliosis or the Mφs/microglia presence at the lesion site. **a**–**h** Immediately following injury, C57BL/6J mice received vehicle, M2 Mφs or IL-13 Mφs. **a**, **b** Quantification of astrogliosis by GFAP intensity analysis (**a**) and Mφ/microglia presence by Iba-1 intensity analysis (**b**) showed no differences between treatment groups. Data are shown as mean ± SEM. *n* = 11–14 mice/group. **c**–**h** Representative images from the spinal cord sections are shown. All analyses were quantified within square areas of 100 μm × 100 μm perilesional placed as indicated in the figure, extending 600 μm rostral to 600 μm caudal from the lesion center (white line). Scale bar = 500 µm. Two-way ANOVA with Bonferroni post hoc test.

**Additional file 5: Figure S4.** IL-13 Mφs are not present at the spinal cord 8 days after transplantation. **a**–**d** Immediately following injury, C57BL/6j mice received vehicle, GFP^+^M2 Mφs or GFP^+^IL-13 Mφs. **a** Quantification of the number of GFP^+^ cells at the injection, perilesional or lesion site after SCI. Data are shown as mean ± SEM. *n* = 3–6 mice/group. **b**–**d** Representative images of the lesion site from the spinal cord sections are shown. GFP^+^ cells are indicated by white arrows. Scale bar = 100 µm. Kruskal–Wallis test with Dunn’s correction.*P < 0.05, and **P < 0.01.

**Additional file 6: Figure S5.** IL-13 Mφ transplantation does not affect the number of Iba-1^+^ cells at the lesion site. **a** Immediately following injury, C57BL/6j mice received vehicle, M2 Mφs or IL-13 Mφs. Quantification of the number of Iba-1^+^ cells at the lesion site showed no differences between vehicle- and IL-13 Mφ-treated mice. Data are shown as mean ± SEM. *n* = 8–9 mice/group. One-way ANOVA with Bonferroni post hoc test. *P < 0.05. *n.s.* not significant.

**Additional file 7: Figure S6.** IL-13 Mφs secrete IL-13 and Arg1 Mφs express Arg1. **a** Mφs were isolated from IL-4R WT BALB/c mice. IL-13 secretion by the IL-13 Mφs was confirmed using ELISA. Data represent mean ± SEM. *n* = 2 independent in vivo experiments. **b**, **c** Mφs were isolated from C57BL/6J mice. Overexpression of Arg1 by the Arg1 Mφs compared to M0 Mφs was confirmed on gene (**b**, qPCR, *n* = 3) and protein (**c**, Western blot, *n* = 1 independent in vivo experiment) level. Data were normalized to M0 Mφs and represent mean ± SEM. Kruskal–Wallis test with Dunn’s correction. *P < 0.05, **P < 0.01, and ***P < 0.001.

**Additional file 8: Figure S7.** IL-13 treatment does not influence cell survival of a murine or human neuroblastoma cell line. **a**, **b** The murine (**a**, Neuro2A) and human (**b**, SH-SY5Y) neuroblastoma cell lines express the alpha-1 subunit of the IL-13R and the alpha unit of the IL-4R as determined via immunocytochemistry. Scale bar = 50 µm. **c**, **d** Neuro2A (**c**) or SH-SY5Y (**d**) cells were treated with different concentrations of rIL-13 (5, 33.3, 50, and 500 ng/ml) for 24, 48 or 72 h. Overall, IL-13 did not affect cell viability of both cell lines determined by an MTT assay. Data were normalized to untreated control and are shown as mean ± SEM. *n* = 4–5. **e**–**h** Neuro2A (**e**, **g**) or SH-SY5Y (**f**, **h**) cells were treated for 48 h with different concentrations of SNAP (100, 200, 400, and 800 µM) to induce cell death. In addition, cells were co-treated (**e**, **f**) or pre-treated (**g**, **h**) for 24 h with different concentrations of rIL-13 (5, 33.3, 50, and 500 ng/ml). Using an MTT assay, neither co-treatment nor pre-treatment with IL-13 protected cells against cell death. Data were normalized to untreated control (= dotted black line) and are shown as mean ± SEM. *n* = 4–5. Kruskal–Wallis test with Dunn’s correction. *P < 0.05, **P < 0.01, and ***P < 0.001.

**Additional file 9: Table S2.** IL-4 secretion. *BDL, below detection limit of ELISA kit (4 pg/ml).

## Data Availability

The data sets used and/or analyzed during the current study are available from the corresponding author on reasonable request.

## Abbreviations

Arg1: Arginase-1
BMS: Basso Mouse Scale
CA: Cornu ammonis
CNS: Central nervous system
CCR: C–C chemokine receptor
Dpi: Days post-injury
FCS: Fetal calf serum
FIZZ1: Found in inflammatory zone 1
GFAP: Glial fibrillary acidic protein
GFP: Green fluorescent protein
hiPSC-NSC: Human-induced pluripotent stem cell-derived neural stem cells
Iba-1: Ionized calcium binding adaptor molecule 1
IL-4Rα KO: Interleukin-4 receptor alpha knockout
IL-13 Mɸs: Interleukin-13 macrophages
iNOS: Inducible nitric oxide synthase
IRF4: Interferon regulatory factor 4
KLF4: Kruppel-like factor 4
KO: Knockout
LCM: L929 conditioned medium
LPS: Lipopolysaccharide
MBP: Myelin basic protein
MHCll: Major histocompatibility complex class ll
MSCs: Mesenchymal stem cells
MTT: 3[4,5-Dimethylthiazol-2-yl]-2,5-diphenyltetrazolium bromide
Mɸs: Macrophages
NF: Neurofilament
NMDA: *N*-methyl-d-aspartic acid
NO: Nitric oxide
OGD: Oxygen–glucose deprivation
ORO: Oil red O
PBS: Phosphate-buffered saline
PFA: Paraformaldehyde
PI: Propidium iodide
rIL-13: Recombinant IL-13
RT: Room temperature
SCI: Spinal cord injury
SEM: Standard error of mean
SNAP: *S*-nitroso-*N*-acetylpenicillamine
TNF-α: Tumor necrosis factor-alpha
WT: Wild type
Ym1: Chitinase-3-like protein 3
