# Supplementary material for: Macrophage-based delivery of interleukin-13 improves functional and histopathological outcomes following spinal cord injury
Source: J Neuroinflammation. 2022 Apr 29;19:102. doi: 10.1186/s12974-022-02458-2 (PMC9052547; doi:10.1186/s12974-022-02458-2)
Supplement: Supplementary file 1 — Additional file 1: Table S1. Primers used for qPCR. [file 12974_2022_2458_MOESM1_ESM.docx]

| **Goal** | **Gene** | **Forward (5’-3’)** | **Reverse (5’-3’)** |
| --- | --- | --- | --- |
| Housekeeping genes | CYCA | GCGTCTCCTTCGAGCTGTT | AAGTCACCACCCTGGCA |
|  | GADPH | GGCCTTCCGTGTTCCTAC | TGTCATCATATCTGGCAGGTT |
|  | HMBS | GATGGGCAACTGTACCTGACTG | CTGGGCTCCTCTTGGAATG |
|  | HPRT | CTCATGGACTGATTATGGACAGGAC | GCAGGTCAGCAAAGAACTTATAGCC |
|  | YHWAZ | GCAACGATGTACTGTCTCTTTTGG | GTCCACAATTCCTTTCTTGTCATC |
| Genes of interest | Arg1 | GTGAAGAACCCACGGTCTGT | GCCAGAGATGCTTCCAACTG |
|  | C5aR | AGGACATGGACCCCATAGATAACA | GCCATCCGCAGGTATGTTAGG |
|  | CCR2 | GGAGCCATACCTGTAAATGCC | TGTCTTCCATTTCCTTTGATTTGT |
|  | CCR5 | ATTCTCCACACCCTGTTTCG | GAATTCCTGGAAGGTGGTCA |
|  | CD38 | ACTGGAGAGCCTACCACGAA | TGGGCCAGGTGTTTGGATTT |
|  | CD86 | GAGCGGGATAGTAACGCTGA | GGCTCTCACTGCCTFCACTC |
|  | CD206 | CTTCGGGCCTTTGGAATAAT | TAGAAGAGCCCTTGGGTTGA |
|  | FIZZ1 | TCCAGCTAACTATCCCTCCACTGT | GGCCCATCTGTTCATAGTCTTGA |
|  | iNOS | CCCTTCAATGGTTGGTACATGG | ACATTGATCTCCGTGACAGCC |
|  | IRF4 | GACCAGTCACACCCAGAAATCCC | GTTCCTGTCACCTGGCAACC |
|  | KLF4 | AACATGCCCGGACTTACAAA | TTCAAGGGAATCCTGGTCTTC |
|  | TNF-α | GTCCCCAAAGGGATGAGAAGT | TTTGCTACGACGTGGGCTAC |
|  | Ym1 | GGGCATACCTTTATCCTGAG | CCACTGAAGTCATCCATGTC |
