# Supplementary figures and images for: Macrophage-based delivery of interleukin-13 improves functional and histopathological outcomes following spinal cord injury
Source: J Neuroinflammation. 2022 Apr 29;19:102. doi: 10.1186/s12974-022-02458-2 (PMC9052547; doi:10.1186/s12974-022-02458-2)

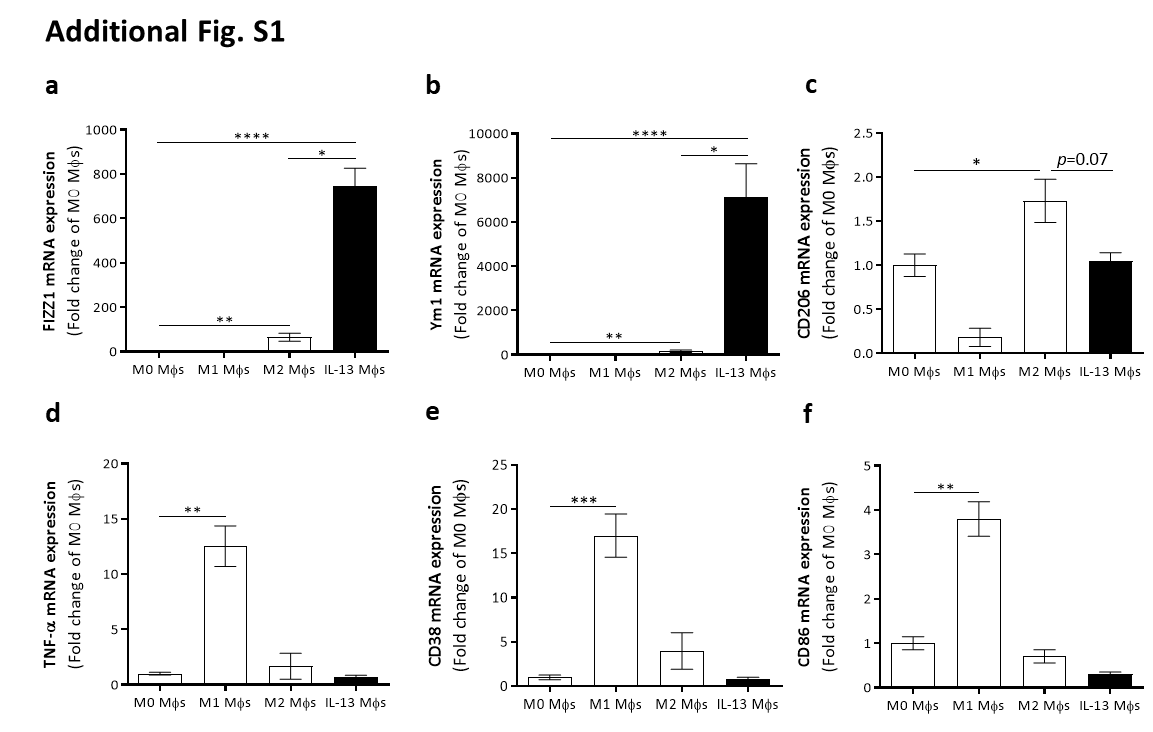

Supplement: Supplementary file 2 — Additional file 2: Figure S1. IL-13 Mφs have an anti-inflammatory phenotype. a–f Mφs were isolated from C57BL/6J mice. qPCR showed that gene expression of the anti-inflammatory markers FIZZ1 (a) and Ym1 (b) were significantly increased in the IL-13 Mφs compared to M0 Mφs, whereas CD206 (c) was not. Pro-inflammatory gene expression of TNF-α (d), CD38 (e), and CD86 (f) were not induced in the IL-13 Mφs. Data were normalized to M0 Mφs and represent mean ± SEM. n = 12–14. Kruskal–Wallis test with Dunn’s correction. *P < 0.05, **P < 0.01, ***P < 0.001, and ****P < 0.0001. [file 12974_2022_2458_MOESM2_ESM.docx]

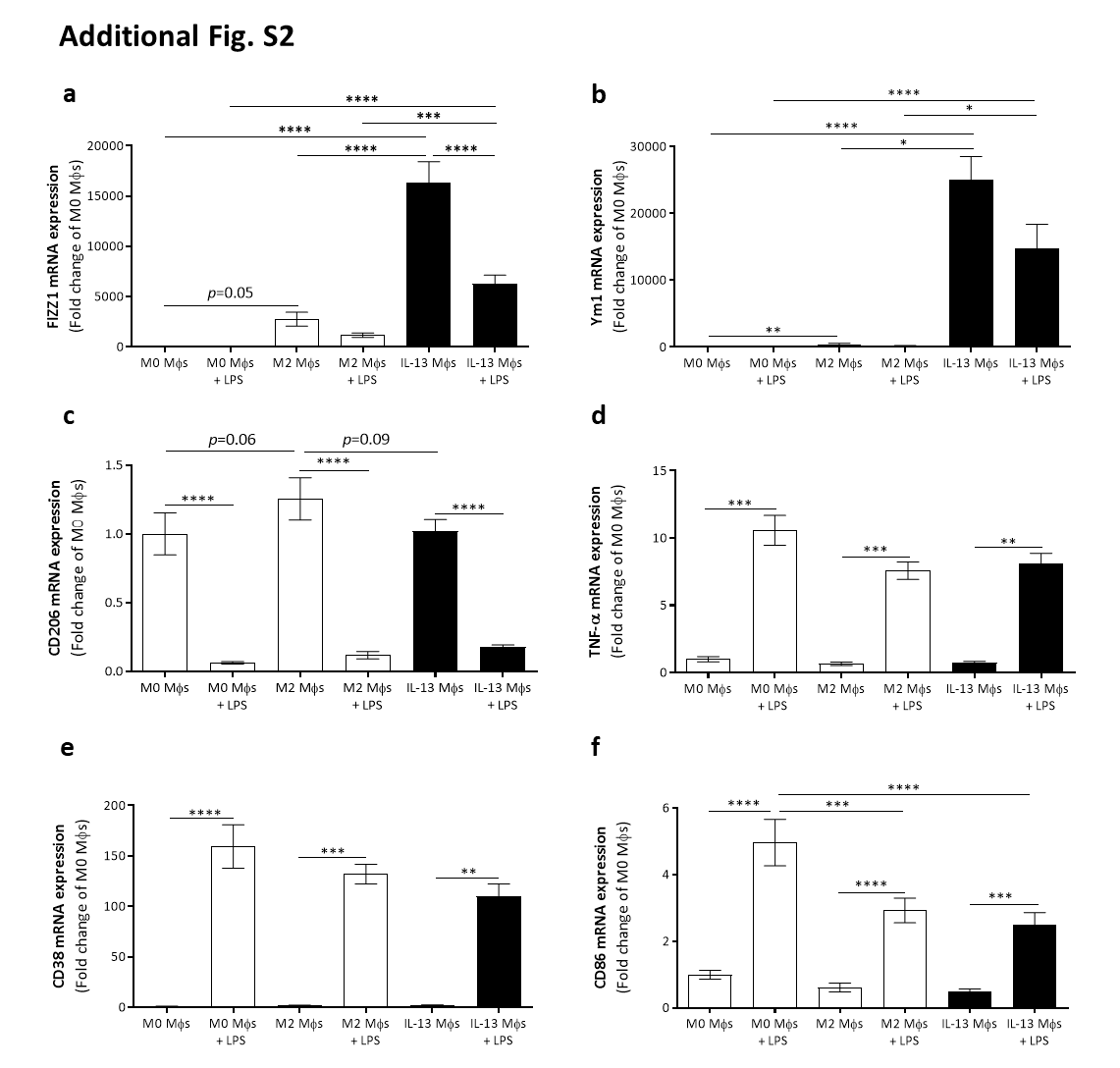

Supplement: Supplementary file 3 — Additional file 3: Figure S2. IL-13 Mφs maintain their anti-inflammatory markers upon LPS stimulation. a–f Mφs were isolated from C57BL/6J mice. M0, M2, and IL-13 Mφs were left unstimulated (control) or were stimulated with LPS for 24 h. a–c qPCR showed that upon incubation with LPS, IL-13 significantly decreased the expression of the anti-inflammatory markers FIZZ1 (a) and CD206 (c) expression, whereas their expression of Ym1 (b) was maintained. Data were normalized to M0 Mφs and represent mean ± SEM. n = 8–9. d–f Pro-inflammatory gene expression of TNF-α (d), CD38 (e), and CD86 (f) were induced in the IL-13 Mφs upon LPS incubation as determined by qPCR. Data were normalized to M0 Mφs and represent mean ± SEM. n = 8–9. Kruskal–Wallis test with Dunn’s correction (b, d, e) or one-way ANOVA with a Bonferroni post hoc test (a, c, f). *P < 0.05, **P < 0.01, ***P < 0.001, and ****P < 0.0001. [file 12974_2022_2458_MOESM3_ESM.docx]

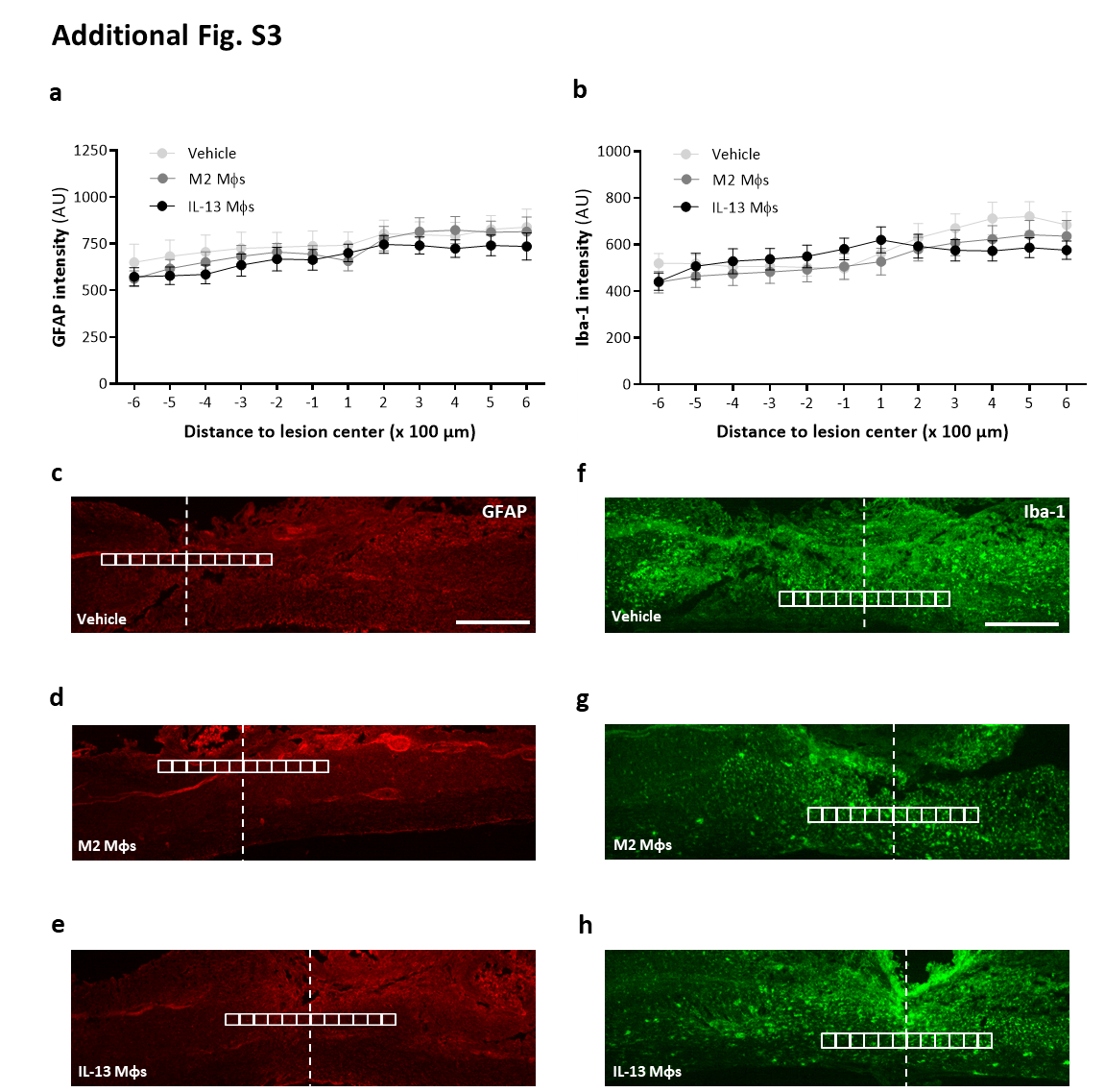

Supplement: Supplementary file 4 — Additional file 4: Figure S3. IL-13 Mφ transplantation does not affect astrogliosis or the Mφs/microglia presence at the lesion site. a–h Immediately following injury, C57BL/6J mice received vehicle, M2 Mφs or IL-13 Mφs. a, b Quantification of astrogliosis by GFAP intensity analysis (a) and Mφ/microglia presence by Iba-1 intensity analysis (b) showed no differences between treatment groups. Data are shown as mean ± SEM. n = 11–14 mice/group. c–h Representative images from the spinal cord sections are shown. All analyses were quantified within square areas of 100 μm × 100 μm perilesional placed as indicated in the figure, extending 600 μm rostral to 600 μm caudal from the lesion center (white line). Scale bar = 500 µm. Two-way ANOVA with Bonferroni post hoc test. [file 12974_2022_2458_MOESM4_ESM.docx]

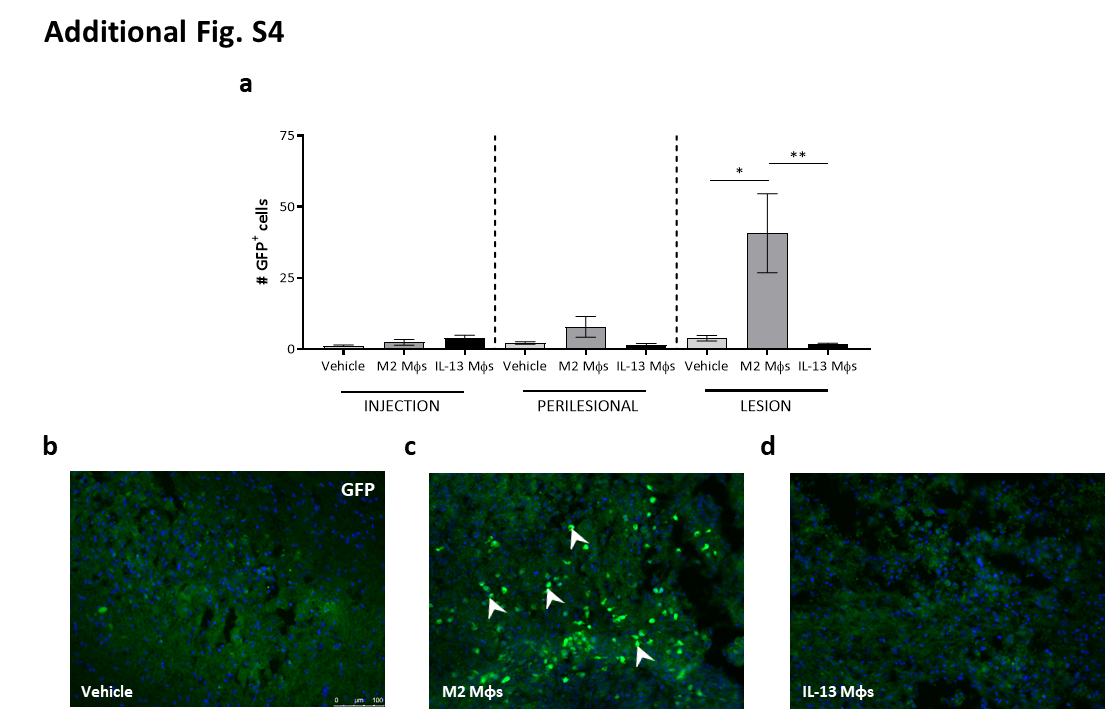

Supplement: Supplementary file 5 — Additional file 5: Figure S4. IL-13 Mφs are not present at the spinal cord 8 days after transplantation. a–d Immediately following injury, C57BL/6j mice received vehicle, GFP+M2 Mφs or GFP+IL-13 Mφs. a Quantification of the number of GFP+ cells at the injection, perilesional or lesion site after SCI. Data are shown as mean ± SEM. n = 3–6 mice/group. b–d Representative images of the lesion site from the spinal cord sections are shown. GFP+ cells are indicated by white arrows. Scale bar = 100 µm. Kruskal–Wallis test with Dunn’s correction.*P < 0.05, and **P < 0.01. [file 12974_2022_2458_MOESM5_ESM.docx]

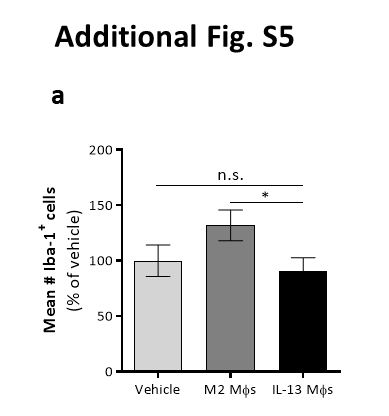

Supplement: Supplementary file 6 — Additional file 6: Figure S5. IL-13 Mφ transplantation does not affect the number of Iba-1+ cells at the lesion site. a Immediately following injury, C57BL/6j mice received vehicle, M2 Mφs or IL-13 Mφs. Quantification of the number of Iba-1+ cells at the lesion site showed no differences between vehicle- and IL-13 Mφ-treated mice. Data are shown as mean ± SEM. n = 8–9 mice/group. One-way ANOVA with Bonferroni post hoc test. *P < 0.05. n.s. not significant. [file 12974_2022_2458_MOESM6_ESM.docx]

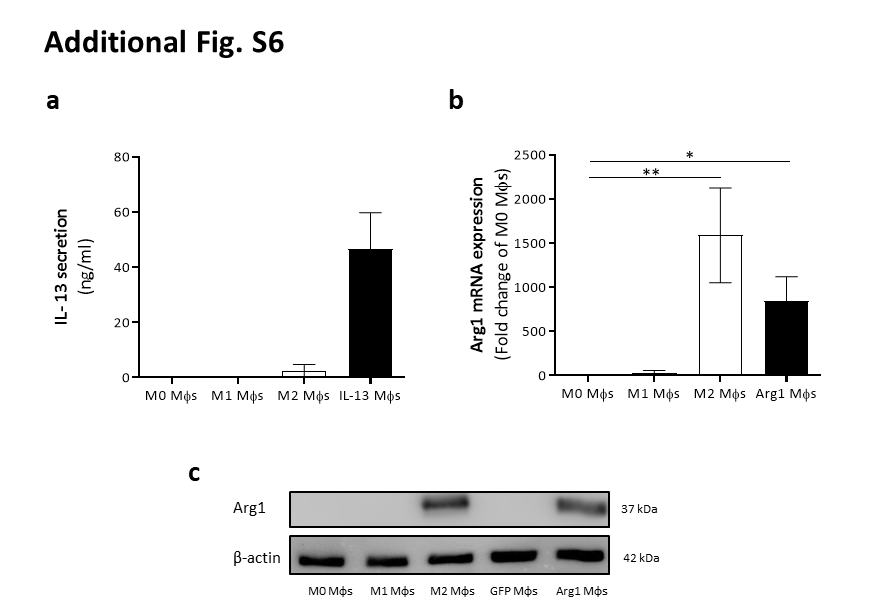


**Original western blot gels:**

- Arg1


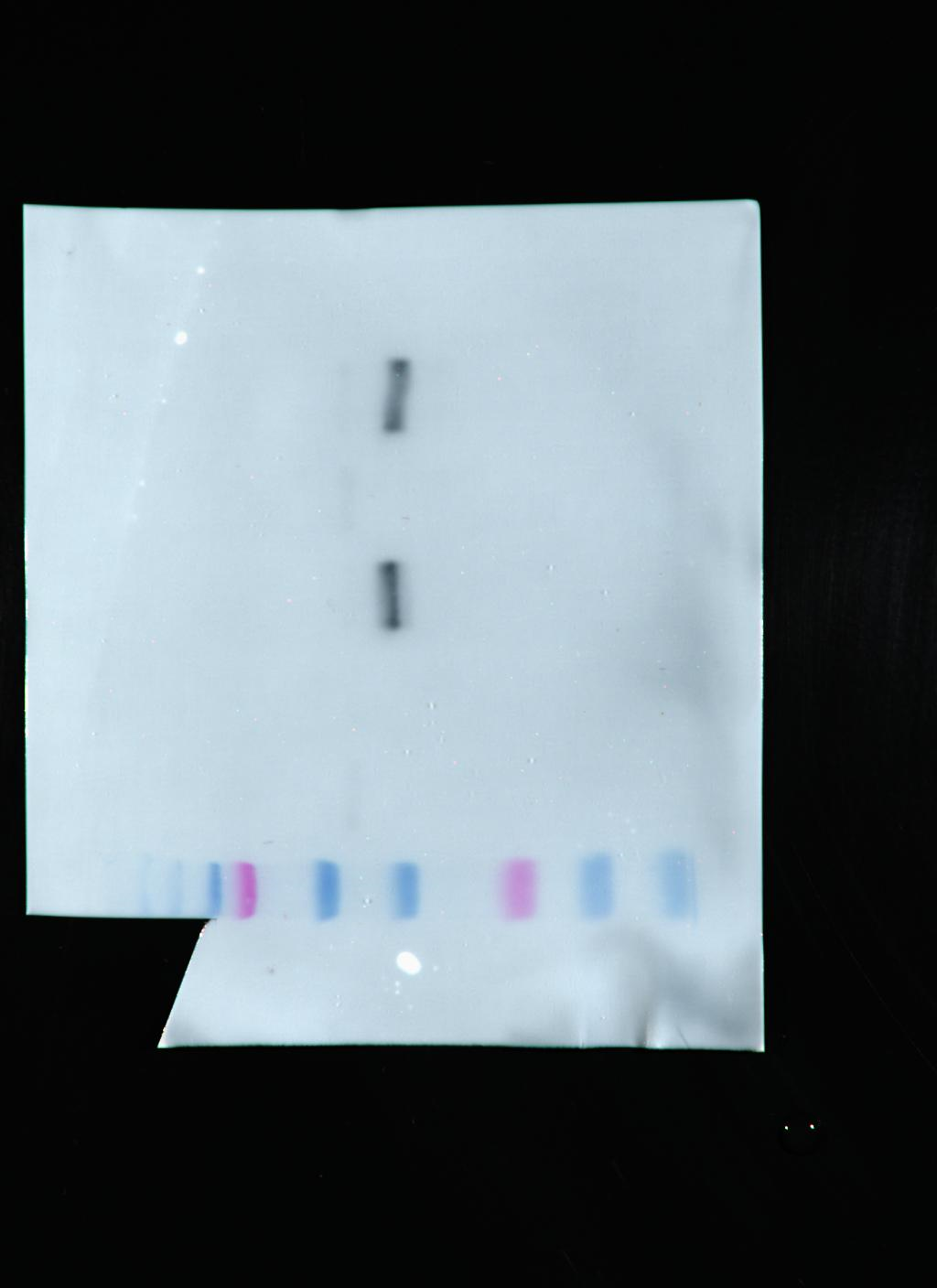


M0 M1 M2 GFP IL-13

Mφs Mφs Mφs Mφs Mφs

- β-actine


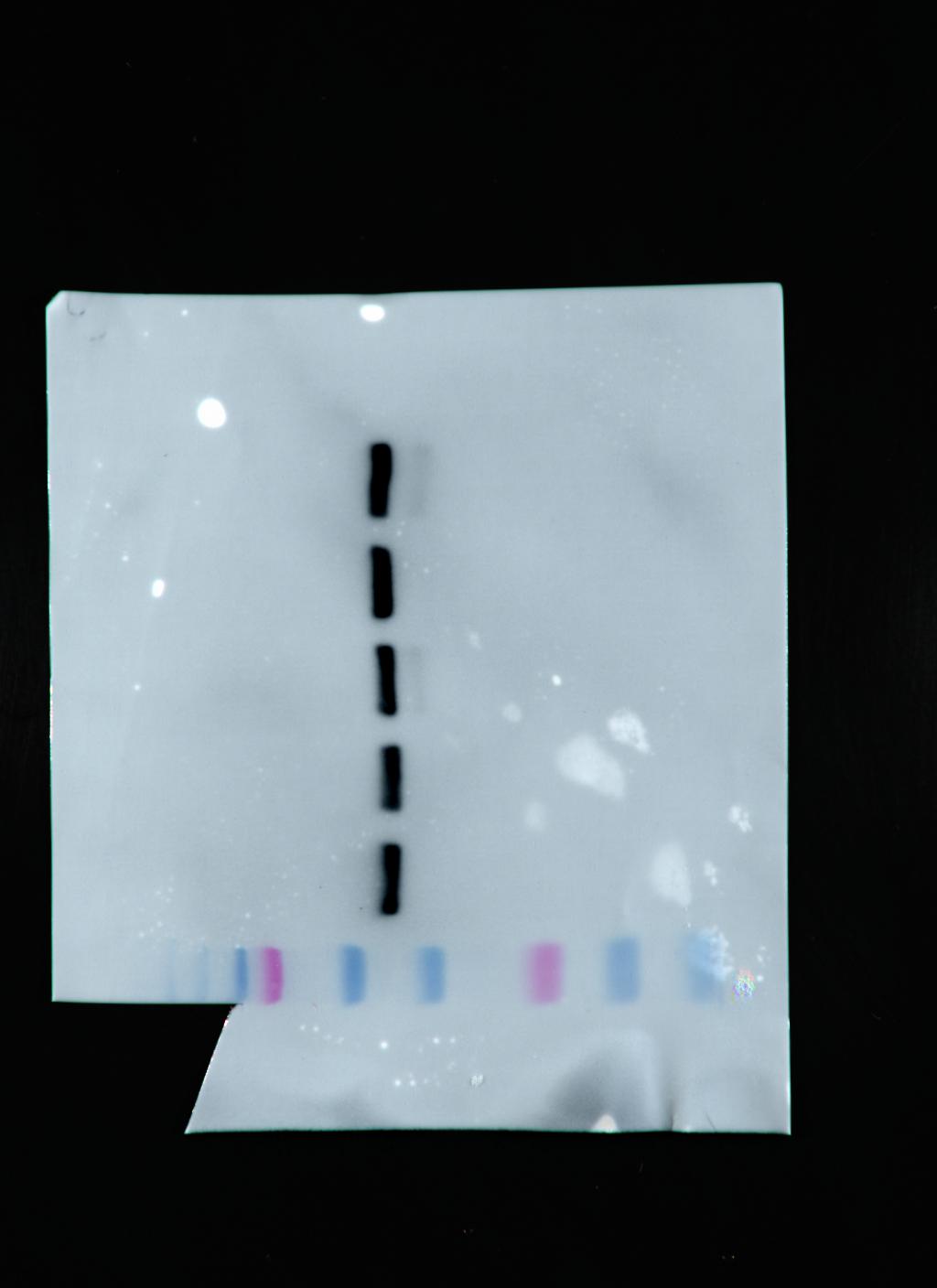


M0 M1 M2 GFP IL-13

Mφs Mφs Mφs Mφs Mφs

Supplement: Supplementary file 7 — Additional file 7: Figure S6. IL-13 Mφs secrete IL-13 and Arg1 Mφs express Arg1. a Mφs were isolated from IL-4R WT BALB/c mice. IL-13 secretion by the IL-13 Mφs was confirmed using ELISA. Data represent mean ± SEM. n = 2 independent in vivo experiments. b, c Mφs were isolated from C57BL/6J mice. Overexpression of Arg1 by the Arg1 Mφs compared to M0 Mφs was confirmed on gene (b, qPCR, n = 3) and protein (c, Western blot, n = 1 independent in vivo experiment) level. Data were normalized to M0 Mφs and represent mean ± SEM. Kruskal–Wallis test with Dunn’s correction. *P < 0.05, **P < 0.01, and ***P < 0.001. [file 12974_2022_2458_MOESM7_ESM.docx]

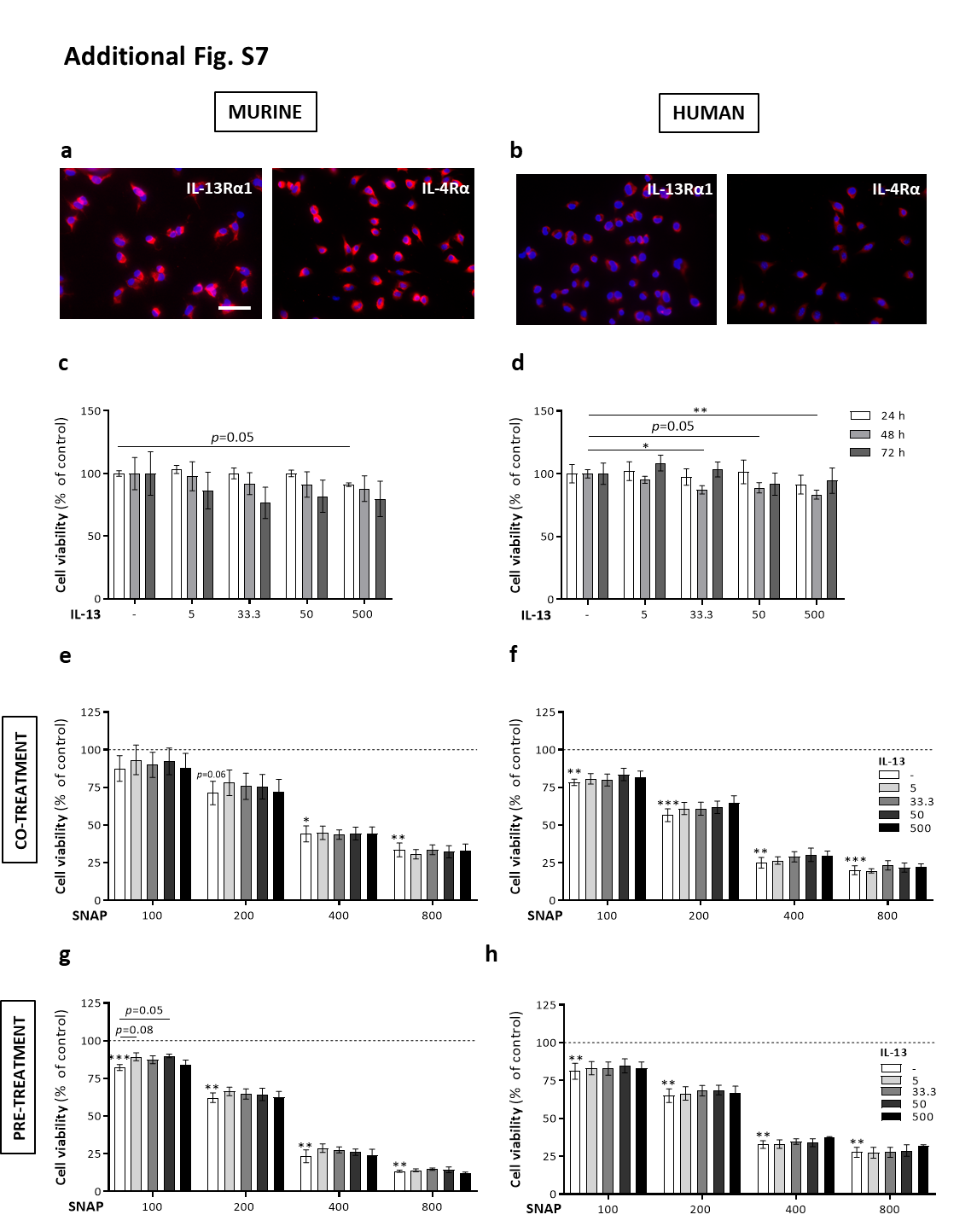

Supplement: Supplementary file 8 — Additional file 8: Figure S7. IL-13 treatment does not influence cell survival of a murine or human neuroblastoma cell line. a, b The murine (a, Neuro2A) and human (b, SH-SY5Y) neuroblastoma cell lines express the alpha-1 subunit of the IL-13R and the alpha unit of the IL-4R as determined via immunocytochemistry. Scale bar = 50 µm. c, d Neuro2A (c) or SH-SY5Y (d) cells were treated with different concentrations of rIL-13 (5, 33.3, 50, and 500 ng/ml) for 24, 48 or 72 h. Overall, IL-13 did not affect cell viability of both cell lines determined by an MTT assay. Data were normalized to untreated control and are shown as mean ± SEM. n = 4–5. e–h Neuro2A (e, g) or SH-SY5Y (f, h) cells were treated for 48 h with different concentrations of SNAP (100, 200, 400, and 800 µM) to induce cell death. In addition, cells were co-treated (e, f) or pre-treated (g, h) for 24 h with different concentrations of rIL-13 (5, 33.3, 50, and 500 ng/ml). Using an MTT assay, neither co-treatment nor pre-treatment with IL-13 protected cells against cell death. Data were normalized to untreated control (= dotted black line) and are shown as mean ± SEM. n = 4–5. Kruskal–Wallis test with Dunn’s correction. *P < 0.05, **P < 0.01, and ***P < 0.001. [file 12974_2022_2458_MOESM8_ESM.docx]
