# Supplementary material for: Macrophage-based delivery of interleukin-13 improves functional and histopathological outcomes following spinal cord injury
Source: J Neuroinflammation. 2022 Apr 29;19:102. doi: 10.1186/s12974-022-02458-2 (PMC9052547; doi:10.1186/s12974-022-02458-2)
Supplement: Supplementary file 9 — Additional file 9: Table S2. IL-4 secretion. *BDL, below detection limit of ELISA kit (4 pg/ml). [file 12974_2022_2458_MOESM9_ESM.docx]

| **Samples** | **OD_450_** | **Concentration** |
| --- | --- | --- |
| Standards | 2.929 | 500 pg/ml |
|  | 1.919 | 250 pg/ml |
|  | 1.123 | 125 pg/ml |
|  | 0.641 | 62.5 pg/ml |
|  | 0.389 | 31.25 pg/ml |
|  | 0.260 | 15.625 pg/ml |
|  | 0.178 | 7.813 pg/ml |
|  | 0.166 | 0 pg/ml |
| M0 Mφs | 0.100 | *BDL |
| M1 Mφs | 0.097 | *BDL |
| M2 Mφs | 0.092 | *BDL |
| IL-13 Mφs | 0.097 | *BDL |
